# The effects of work on cognitive functions: a systematic review

**DOI:** 10.3389/fpsyg.2024.1351625

**Published:** 2024-05-09

**Authors:** Pasquale Bufano, Cristina Di Tecco, Alice Fattori, Teresa Barnini, Anna Comotti, Catalina Ciocan, Luca Ferrari, Francesca Mastorci, Marco Laurino, Matteo Bonzini

**Affiliations:** ^1^Institute of Clinical Physiology, National Research Council, Pisa, Italy; ^2^Department of Occupational and Environmental Medicine, Epidemiology and Hygiene, Italian Workers' Compensation Authority (INAIL), Rome, Italy; ^3^Occupational Medicine Unit, Foundation IRCCS Ca' Granda Ospedale Maggiore Policlinico, Milan, Italy; ^4^Department of Clinical Science and Community Health, University of Milan, Milan, Italy; ^5^Department of Public Health and Pediatrics, University of Turin, Turin, Italy

**Keywords:** cognitive functions, aging workforce, occupational stress, shift work, sedentary work, cognitive impairment

## Abstract

**Introduction:**

Cognitive functions play a crucial role in individual’s life since they represent the mental abilities necessary to perform any activity. During working life, having healthy cognitive functioning is essential for the proper performance of work, but it is especially crucial for preserving cognitive abilities and thus ensuring healthy cognitive aging after retirement. The aim of this paper was to systematically review the scientific literature related to the effects of work on cognitive functions to assess which work-related factors most adversely affect them.

**Method:**

We queried the PubMed and Scopus electronic databases, in February 2023, according to the PRISMA guidelines (PROSPERO ID number = CRD42023439172), and articles were included if they met all the inclusion criteria and survived a quality assessment. From an initial pool of 61,781 papers, we retained a final sample of 64 articles, which were divided into 5 categories based on work-related factors: shift work (*n* = 39), sedentary work (*n* = 7), occupational stress (*n* = 12), prolonged working hours (*n* = 3), and expertise (*n* = 3).

**Results:**

The results showed that shift work, occupational stress, and, probably, prolonged working hours have detrimental effects on cognitive functioning; instead, results related to sedentary work and expertise on cognitive functions are inconclusive and extremely miscellaneous.

**Discussion:**

Therefore, workplace health and well-being promotion should consider reducing or rescheduling night shift, the creation of less demanding and more resourceful work environments and the use of micro-breaks to preserve workers’ cognitive functioning both before and after retirement.

**Systematic review registration:**

https://www.crd.york.ac.uk/prospero/display_record.php?ID=CRD42023439172, identifier CRD42023439172.

## 1 Introduction

Cognitive functions play a crucial role in the daily life of every human being because they represent the basic mental abilities for performing any activity. They include attention, memory and learning, perceptual motor function, executive functions, and language; each of these allows human beings to perceive the world around them and to make decisions, but also to perform from the simplest task, such as remembering a telephone number or brushing teeth, to more complex ones such as reading a book or using the internet. Among the various activities that we are able to perform with the support of cognitive functions is clearly included the execution of any kind of work. During working life, having healthy cognitive functioning is essential for the proper performance of work, but it is especially crucial for preserving cognitive abilities and thus ensuring healthy cognitive aging after retirement (Cohen et al., 2019). In everyday life, there are a lot of different factors that can contribute to the preservation of adequate cognitive abilities, such as physical activity and social engagement (Ihle et al., 2015), mental activities (e.g., taking courses, learning new languages) or cognitively stimulating leisure activities (e.g., go to the theatre) (Hultsch et al., 1993; Schooler and Mulatu, 2001), and it is well known that even a high cognitive level of one’s job leads to cognitive enrichment (Stern et al., 1995; Schooler et al., 1999), in the same way as all other variables. On the contrary, however, there are also many factors that negatively impact cognitive functions such as, for example, prolonged exposure to stress leading to the loss of neurons, particularly in the hippocampus, which is altered by the increase in glucocorticoids and results in cognitive impairment (McEwen and Sapolsky, 1995), sleep problems such as insomnia can induce cognitive impairment (Brownlow et al., 2020) but even acute states of sleep deprivation can trigger deterioration in one or more cognitive functions (Wang et al., 2021; Bufano et al., 2023), and sedentary behaviour is associated with lower cognitive performance (Falck et al., 2017). All these factors, both positive and negative, may be intrinsic in different types of work and may contribute to both adequate preservation of cognitive functions and cognitive impairment. Therefore, the study of how work can have an impact on cognitive function, both in a positive way and, above all, in a preventive health perspective, in a negative way, has become a priority in the field of prevention and healthy aging, as shown by the growing interest of the European community (Salazar-Xirinachs, 2012; Vendramin et al., 2012; Belin et al., 2016) and latest research on the topic (Vives et al., 2018; Rinsky-Halivni et al., 2022; Bonzini et al., 2023).

If we look at cognitive aging from the perspective of normative aging, there are three main theories explaining the effects of work on cognitive functions and take into account both the level of mental activity experienced during working life and the type of working environment encountered by the worker. The primary theory, which currently guides the line of research, is the *“use-it-or-lose-it”* hypothesis (Hultsch et al., 1999; Salthouse, 2006, 2016), according to which the individual level of cognitive functioning depends on two mechanisms: *differential preservation* and *preserved differentiation.* For the first one, individuals who are mentally active tends to preserve their cognitive functions more than those who are not mentally active; the second one postulates that individuals with higher levels of cognitive functioning during life retain these beneficial levels as they age. There are two other prominent theories: *“the Brain or Cognitive reserve hypothesis”* (Mortimer and Graves, 1993; Stern, 2002; Fratiglioni and Wang, 2007) and *“Schooler’s theory of environmental influences on cognitive functioning”* (Schooler, 1984, 1990). The cognitive reserve hypothesis states that people who attend mentally stimulating environments will have improved neuronal development, resulting in increased cognitive reserve. This cognitive reserve could be critical in facing neuronal insults and cognitive decline, increasing individual resilience, and thus leading to healthier aging (Mortimer and Graves, 1993; Stern, 2002; Fratiglioni and Wang, 2007). The Schooler’s theory holds that complex environments that promotes cognitive effort lead people to develop their intellect and apply new cognitive processes, thereby improving cognitive functioning. Conversely, if the environment do not stimulate high levels of cognitive functioning, intellectual abilities will not be maintained and there will be a decrease in cognitive functioning (Schooler, 1984, 1990). All these theoretical frameworks follow the same line: by stimulating cognitive functions in a positive way, either through mentally active jobs or enriched working environments, cognitive functions are preserved and protected, and people move toward healthier cognitive aging.

On the other hand, however, these theories remain valid and applicable as long as we talk about normative aging, since there are several factors intrinsic to various types of work that can have a negative impact on cognitive functions, causing short-term cognitive impairment and early cognitive decline after retirement. Referring to the negative factors mentioned above (i.e., sleep disturbances, stress, and sedentary behaviour), there are several studies in the literature that relate them to different types of work and show the negative effects they could have on cognitive functioning. Shift work (i.e., an employment practice designed to keep a service or production line operational at all times, even during the night) is certainly linked to sleep disturbances that can induce cognitive impairment, but also to cardiovascular diseases (Kantermann et al., 2012) which can lead to the same outcome (Picano et al., 2014). Highly demanding jobs that induce work-related stress (i.e., a response that people may have when exposed to job demands and pressures that do not match their knowledge and skills) may increase the risk of dementia (Andel et al., 2012), and sedentary behaviour [i.e., any waking behaviour characterized by an energy expenditure ≤1.5 metabolic equivalents (METs), while in a sitting, reclining, or lying posture (Marconcin et al., 2021)], caused by office work has as a potential outcome cognitive impairment (Park et al., 2020), although its effects are often mixed (Magnon et al., 2018).

We identified some work-related factors that can adversely affect cognitive functions and lead to cognitive impairment and probable pathological aging after retirement, but they are probably not the only ones; a comprehensive understanding of what all these factors are would be very important both from a prevention perspective and from a care perspective, with the aim of protecting their cognitive health and trying to ensure healthy cognitive aging.

Therefore, to achieve this goal and obtain a comprehensive overview of work-related factors influencing cognitive functions, our idea is to gather all the scientific literature on the topic through a systematic review using both, specific keywords related to the above-mentioned factors that negatively adverse cognitive functions, and generic keywords to try to broaden the search and discover other factors that may also exist.

## 2 Materials and methods

### 2.1 Study design and search strategy

We conducted this systematic review following the Preferred Reporting Items for Systematic Reviews and Meta-Analyses (PRISMA) guidelines (Moher et al., 2009; Page et al., 2021). PRISMA comprises a 27-item checklist to ensure and promote the quality of systematic reviews; this checklist is reported in Supplementary Tables S1, S3 (for abstract checklist). The protocol employed in the current systematic review has been submitted for registration (ID number = CRD42023439172) to the international prospective register for systematic reviews database (PROSPERO. Available online https://www.crd.york.ac.uk/prospero/display_record.php?ID=CRD42023439172; accessed on July 06, 2023). The retrieval process consisted of three phases.

### 2.2 Systematic search phases

#### 2.2.1 Preliminary research and definition of keywords

During the first phase, started in February 2023, we carried out preliminary research in absence of specific keywords based on the review of Fisher et al. (2017), to define search criteria and identify the specific keywords to be used. Before defining the final list of keywords, we started from a very large list that, when entered into search engines, produced a massive number of results. We therefore removed nonspecific keywords and, on the fourth attempt, based on our research question, defined specific keywords (see Table 1) related to two domains: “Work” and “Cognitive Functions,” for inclusion in search engines to conduct the systematic research.

**Table 1:** List of selected keywords.

Keywords	
Work	Cognitive functions
“day work”	“cognitive assessment”
“day workers”	“cognitive domain”
“employee”	“cognitive function”
“employees”	“cognitive functioning”
“employment”	“cognitive functions”
“job”	“executive assessment”
“jobs”	“executive domain”
“labor”	“executive function”
“occupational stress”	“executive functioning”
“occupational”	“executive functions”
“sedentary behaviour”	“neuropsychological assessment”
“sedentary behaviour”	
“sedentary work”	
“shift work”	
“shift workers”	
“work”	
“worker”	
“workers”	
“workplace”	

#### 2.2.2 Systematic search and definition of PICOS

The second phase, conducted in February 2023, consisted of a systematic search among titles, abstracts, and keywords of scientific papers, using the electronic databases PubMed and Scopus, based on the selected keywords, properly combined through the Boolean operators “AND” and “OR”; no temporal restriction was set, so all articles published up to February 2023 in Italian and English were included in the systematic search. The search strategy, including all keywords used and the number of studies found from each database, was analytically reported in Table 2.

**Table 2:** Search strategy.

Database	Steps	Query	Research in	Items found
PubMed	#1	“day work”[Title/Abstract] OR “day workers”[Title/Abstract] OR “employee”[Title/Abstract] OR “employees”[Title/Abstract] OR “employment”[Title/Abstract] OR “job”[Title/Abstract] OR “jobs”[Title/Abstract] OR “labor”[Title/Abstract] OR “occupational stress”[Title/Abstract] OR “occupational”[Title/Abstract] OR “sedentary behaviour”[Title/Abstract] OR “sedentary behaviour”[Title/Abstract] OR “sedentary work”[Title/Abstract] OR “shift work”[Title/Abstract] OR “shift workers”[Title/Abstract] OR “work”[Title/Abstract] OR “worker”[Title/Abstract] OR “workers”[Title/Abstract] OR “workplace”[Title/Abstract] OR “work-place”[Title/Abstract]	Title/Abstract	1,714,613
	#2	“cognition”[Title/Abstract] OR “cognitive assessment”[Title/Abstract] OR “cognitive domain”[Title/Abstract] OR “cognitive function”[Title/Abstract] OR “cognitive functioning”[Title/Abstract] OR “cognitive functions”[Title/Abstract] OR “executive assessment”[Title/Abstract] OR “executive domain”[Title/Abstract] OR “executive function”[Title/Abstract] OR “executive functioning”[Title/Abstract] OR “executive functions”[Title/Abstract] OR “neuropsychological assessment”[Title/Abstract]	Title/Abstract	195,172
	#3	Combine #1 AND #2		13,963
	#4	Limit to (English) and (Italian)		13,455
Scopus	#1	TITLE-ABS-KEY (“day work” OR “day workers” OR employee OR employees OR employment OR job OR jobs OR labor OR “occupational stress” OR occupational OR “sedentary behavior” OR “sedentary behaviour” OR “sedentary work” OR “shift work” OR “shift workers” OR work OR worker OR workers OR workplace OR “work-place”)	Title/Abstract/Keywords	7,643,852
	#2	TITLE-ABS-KEY (cognition OR “cognitive assessment” OR “cognitive domain” OR “cognitive function” OR “cognitive functioning” OR “cognitive functions” OR “executive assessment” OR “executive domain” OR “executive function” OR “executive functioning” OR “executive functions” OR “neuropsychological assessment”)	Title/Abstract/Keywords	525,089
	#3	Combine #1 AND #2		51,146
	#4	Limit to (English) and (Italian)		48,066

The final selection of papers for inclusion was carried out according to the Population, Intervention, Comparison, Outcomes, and Study Design (PICOS) worksheet (Moher et al., 2009; Page et al., 2021), summarised in Table 3. We defined the following PICOS criteria: Population, we chose “Healthy Workers (or not diagnosed)” as the inclusion criteria and “Clinical based population” as exclusion criteria because we aimed to avoid including populations in which, an organic disease or mental disorder could affect cognitive functions by generating a bias in the assessment; Interventions, we chose as inclusion criteria “Assessment of cognitive function without chemical/ or neurotoxic, or physical hazard exposure” to avoid including jobs where the worker’s exposure to various substances could create a bias in the assessment of cognitive function; Comparisons, we chose as inclusion criteria “Control Group (Between or Within design)” in order to have a comparison between two populations working different jobs (e.g., shift work vs. non-shift work), or between the same population during different shifts (night shift work vs. day shift work), in order to see if there was a variation in cognitive functioning due to the factor the study was investigating; Outcomes, we chose as inclusion criteria “Change or non-change of cognitive function according to different conditions,” to have a real evaluation of cognitive functioning through the use of cognitive test, thus studies with a quantitative measure of cognitive functioning; Study Design, we chose “Review, Scoping Review, Narrative Review, Systematic Review, Meta-Analysis, Editorial, Book, Case Report, Conference Review, and Conference Paper” as exclusion criteria following the guidelines to carry out a Systematic Review.

**Table 3:** Population, intervention, comparison, outcomes and study design (PICOS) worksheet.

Parameters	Inclusion criteria	Exclusion criteria
Participants	Healthy workers (or not diagnosed)	Clinical based population
Interventions	Assessment of cognitive function without chemical/ or neurotoxic, or physical hazard exposure	Assessment of cognitive function with chemical/ or neurotoxic, or physical hazard exposure
Comparisons	Control group (between or within design)	Others
Outcomes	Quantitative change or non-change of cognitive function according to different conditions	Others
Study design	Original studies in English or Italian language	Review, Scoping Review, Narrative Review, Systematic Review, Meta-Analysis, Editorial, Book, Case Report, Conference Review, and Conference Paper

#### 2.2.3 Application of PICOS study design exclusion criteria

The final phase consisted of a first step in which, following PICOS criteria related to the Study Design section, we excluded all reviews, both narrative and systematic ones, meta-analyses, and conference papers, to substantially reduce the number of included studies.

#### 2.2.4 Title and abstract selection

By reading the titles and abstract we excluded all studies that did not match with the research question.

#### 2.2.5 Full-text selection according to PICOS criteria

Finally, we included in the systematic review only clinical or epidemiological studies that investigated the effects of work on cognitive functions in groups of workers. The included papers were read thoroughly to obtain the data of our interest.

#### 2.2.6 Synthesis method

The included papers were clustered according to the work-related factors that may affect cognitive functions and then, for each study, we extracted all the information shown in Tables 4–8 as described next:

**Table 4:** Shift work and cognitive function: retrieved studies and their main outcomes.

Shift work and cognitive							
Study	Country (region)	Goal of the study	Sample	Cognitive domains investigated	Cognitive functions assessed for each domain	Cognitive tests used for each function	Synthesis of main results
Abdelhamid et al. (2020)	Cairo, Egypt	Evaluate and compare differences in cognitive function of anaesthesia residents before and after the 24 h work shift.	*N* = 50 anaesthesia residents	Attention Executive functions	Sustained attention and divided attention Processing speed	Psychomotor vigilance task, trial making test B Trial making test A	Sleep deprivation in night shifts increases the day-time sleepiness and affects the anaesthesiologist’ cognitive processes, such as reaction time, alertness, rapid problem solving, psychomotor skills, attention, mental flexibility, and executive functions.
Adams and Venter (2020)	Stellenbosch, South Africa	Quantify the effect of shift work on multiple cognitive function domains in anaesthesiology trainees.	*N* = 29 anaesthesia trainees	Perceptual-motor function Attention Memory/learning Executive functions	Psychomotor function Selective attention Visual learning Working memory	Detection Identification One card learning One back speed The assessment was made through CogState computerized test battery.	Fatigue in anaesthesiology trainees after a 14-h night shift results in a decline in reaction time in the cognitive domains of psychomotor function and attention.
An et al. (2022)	Sichuan, West China	Explore the effects of different shift work on cognitive and executive performance in a real clinical environment among nurses.	*N* = 18 nurses	Attention	Selective attention	Stroop test	Study showed that increased fatigue was found in nurses after day and evening shifts, and shift work can affect the reaction time after the evening shift.
Anderson et al. (2012)	Boston, USA	Quantify the time course of neurobehavioral deterioration due to repeated exposure to 24-to 30 h shift during a 3-week residency rotation.	*N* = 34 postgraduate year one physicians	Attention	Sustained attention	Psychomotor vigilance test	Chronic sleep deficiency caused progressive degradation in residents’ neurobehavioral performance and exacerbated the effects of acute sleep loss inherent in the 24- to 30-h extended duration work shifts that are commonly used in resident schedules.
Athar et al. (2020)	Tabriz, Iran	Investigate the influence of shift work on the executive functions.	*N* = 60 shift workers of university security staff: 30-day workers 30-night workers	Memory Executive functions Attention	Visuo-spatial memory Cognitive flexibility Sustained attention	Corsi block-tapping test Berg’s card sorting task Continuous performance task	Shift work impaired executive functions (EF). These findings are related to shift workers’ poorer sleep and its detrimental effects on areas of the brain, which are critical for EF, such as the prefrontal area. Results suggest the evaluation and implication of practices and policies to assuage the consequences of working in shifts.
Benítez-Provedo et al. (2022)	Madrid, Spain	Evaluate the change of different cognitive functions after a guard shift in physicians.	*N* = 30 physicians	Executive functions Memory/learning Attention Perceptual-motor function	Processing speed Short-term memory, semantic and phonemic fluency Visuospatial function, divided attention	Symbol digit test, trail making test A Digit span and free and cued selective reminding test, free recall, S-P words Visual orientation and space perception test, trail making test B	The realization of guard shift was associated with a lower yield in several cognitive functions, especially in tasks related to executive function and attention.
Chang et al. (2011)	Taiwan	Compare cognitive performance at the time of maximum fatigue (3–4 am on the last night shift of the rotation) between nurses working two, three, and four consecutive night shifts.	*N* = 62 nurses	Executive functions Attention	Cognitive flexibility Sustained attention, selective attention	Wisconsin card sorting test Digital symbol substitution test and symbol searching test, Taiwan university attention test	Greater impairment of perceptual and motor ability was seen among subjects who worked two consecutive night shifts compared with those who worked four consecutive night shifts.
Chang et al. (2013a)	Taiwan	Explore changes in cognitive functions, sleep propensity, and sleep-related hormones in the daytime of nurses working on fast rotating shifts.	*N* = 20 nurses	Executive functions Attention	Cognitive flexibility Sustained attention, selective attention	Wisconsin card sorting test Digital symbol substitution test and symbol searching test, Taiwan university attention test	Nurses working on fast rotating shifts overestimate the cognitive functions and capacity of maintaining wakefulness following daytime sleep restriction. Attention performance depended on the attentive load requirement and was possibly related to TSH level.
Chang et al. (2013b)	Taiwan	Explore changes in cognitive function, sleep propensity, and sleep-related hormones in the daytime of nurses working after one block of fast forward rotating shift work (2 days, 2 evenings, and 2 nights).	*N* = 20 nurses	Executive functions Attention	Cognitive flexibility Sustained attention, selective attention	Wisconsin card sorting test Digital symbol substitution test and symbol searching test, Taiwan university attention test	There was no change in sleep propensity in the daytime after one block of rotating shift work. An attempt to preserve daytime alertness was also related to maintaining neuropsychological performance. Maintaining this ability was related to thyrotropin and age, and this cognition required a high attentive load.
Deary and Tait (1987)	Edinburgh, Ireland	Evaluate of effect of sleep disruption on cognitive performance and mood in medical house officers.	*N* = 12 medical house officers	Memory/language Executive functions Attention	Short-term memory, logical memory Processing speed Backword counting	Digit span, logical memory test-delay and immediate Information processing The serial 13 s	Though loss of sleep and long hours of work have an effect on memory and mood, the individual differences among doctors are the main source of the variance in performance of tasks.
Esmaily et al. (2022)	Isfahan, Iran	Investigate the relationship between shift work and cognitive performance in nurses.	*N* = 35 female nurses	Executive functions Attention	Working memory Selective attention	Wechsler scale Stroop test	Study showed that shift work can affect some aspects of cognitive function (working memory and attention) in nurses, and this effect was more prominent after a night shift.
Griffiths et al. (2006)	Victoria, Australia	Characterize the cognitive performance of anaesthetic registrars before and after a series of night shifts.	*N* = 9 full-time anaesthetic trainees	Attention Executive functions Memory/learning	Sustained attention Decision making, monitoring Immediate memory, learning	Detection Identification, Monitoring Choice task, learning The assessment was made through CogState computerized test battery.	There was a statistically significant deterioration in speed of performance for detection and identification tasks at the end of night shift as the week progressed. Anaesthetic registrars demonstrate a significant decline in cognitive performance after a series of night shifts.
Haidarimoghadam et al. (2017)	Hamedan, Iran	Evaluate the effects of consecutive night shifts (CNS) and shift length on cognitive performance and sleepiness.	*N* = 30 control room operators	Executive functions Attention	Working memory, continuous performance test Vigilance	NBACK, CPT Simple reaction time test	The correct responses and response times of working memory were reduced (*p* = 0.001), while intentional errors and sleepiness increased during the shift work (*p* = 0.001). CNS had a significant impact on reaction time and commission errors (*p* = 0.001).
James et al. (2021)	Northwestern United States	Determine the extent of changes in cognition and sleepiness in nurses working three consecutives 12 h shifts.	*N* = 94 nurses working in 12 h shifts: 44 Day shift 49 Night shift	Attention	Sustained attention	Psychomotor vigilance test	There was a significant main effect of shift duty on psychomotor vigilance mean RTs and on the number of transformed psychomotor lapses.
Kazemi et al. (2016)	Hamedan, Iran	Examine cognitive performance, sleepiness, and sleep quality among petrochemical control room shift workers.	*N* = 60 petrochemical control room shift workers	Executive functions Attention	Working memory, continuous performance test Vigilance	NBACK, CPT Simple reaction time test	Long working hours per shift result in fatigue, irregularities in the circadian rhythm and the cycle of sleep, induced cognitive performance decline at the end of both day and night shifts, and increased sleepiness in night shift.
Kazemi et al. (2018)	Hamedan, Iran	Measure cognitive performance, melatonin rhythms, and sleep after different consecutive night shifts (7 vs. 4) among control room operators	*N* = 60 control room operators (CORs)	Executive functions	Working memory, continuous performance test	NBACK, CPT	Individuals who worked 7 consecutive night shifts had a significantly better cognitive performance and sleep quality than those who worked 4 consecutive night shifts. An appropriate number of consecutive night shifts in a rotating shift system should be planned with the ultimate aim of improving CROs performance/alertness and enhancing safety.
Lingenfelser et al. (1994)	Tubingen, Germany	Evaluate task-specific cognitive status and emotional condition after night duty	*N* = 40 young hospital doctors: 27 men 13 women	Executive functions Memory Attention	Processing speed Free recall Sustained attention, selective attention	Number connection test Things-to-do list Vienna reaction timer, Stroop test	Cognitive function and mood status of young hospital doctors after a night on call decrease considerably.
Maltese et al. (2016)	Marseille, France	Assess the impact of an intensive care unit (ICU) night shift on the cognitive performance of a group of intensivists.	*N* = 51 intensivists	Executive functions Memory	Perceptual reasoning capacity, processing speed, cognitive flexibility Short-term memory	Picture completion, coding subtest, Wisconsin sorting card test Digit span	The cognitive abilities of intensivists were significantly altered following a night shift in the ICU.
Niu et al. (2013)	Taiwan	Investigate selective attention levels of nursing staff on different shifts.	*N* = 62 nursing staff	Attention	Selective attention	D2 Test	Inadequate sleep and a state of somnolence adversely affected the attention and operation speed of work among night-shift workers.
Orton and Gruzelier (1989)	London, UK	Investigate cognitive effects of night duty on young doctors.	*N* = 20 junior doctors	Executive functions Attention Perceptual-motor function	Decision making Vigilance Tactile discrimination	Choice reaction time Vigilance reaction time Haptic sorting task	Continuous working may adversely affect the cognitive function and mood.
Özdemir et al. (2013)	Van, Turkey	Investigate the effects of shift work on participants’ cognitive functions in terms of memory, attention, and learning.	*N* = 90 health care workers: 45-night shift 45-day shift	Executive functions Attention Memory	Mental control Concentration, selective attention Visual and verbal memory, delayed recall, general memory	Wechsler memory scale (WMS-R) WMS-R, Stroop color word interference WMS-R, auditory verbal learning test	Findings suggest that night shift work may result in significantly poorer cognitive performance, particularly working memory.
Peng et al. (2022)	Beijing, China	Explore the effect of night shift work on the decision-making competence and performance of the Iowa Gambling Task (IGT)	*N* = 107 female nurses	Executive functions	Decision making	Iowa gambling task	The decrease in decision-making competence which was related with poor decision-making due to nightshift work.
Persico et al. (2018)	Marseille, France	Evaluate the cognitive performance of emergency physicians after a night shift of 14 h (H14) and after a work shift of 24 h (H24) and to compare it with tests performed after a rest night at home (H0).	*N* = 40 emergency physicians: 19 staff physicians 21 residents	Memory Executive functions	Working Memory Processing Speed, Perceptual reasoning, and Cognitive flexibility	WAIS WAIS, WAIS, and WSCT	The cognitive abilities of emergency physicians were significantly altered after a 24-h shift, whereas they were not significantly different from the rested (H0) condition after a 14-h night shift.
Petru et al. (2005)	Munich, Germany	Clarify whether cognitive and psychomotor performance, which are important for occupational and traffic safety, are impaired by working permanent night shifts (NSs) compared with early–late two shifts (TSs)	*N* = 44 automobile workers: 20 working two early late shifts 24 working night shifts	Attention	Selective attention, vigilance, visual alertness, attention deficits	D2, Vienna reaction test, line-marking test, Vienna determination test	The results of this study indicate that—if chosen voluntarily—working NSs has no immediate negative effects on cognitive and psychomotor performance when compared with working TSs.
Prasad et al., 2021	Puducherry, India	Evaluate the changes in cognitive and psychomotor functions in the anaesthesia residents after 6 and 12 h of continuous work.	*N* = 60 anaesthesia residents	Attention Memory Executive functions	Sustained attention Spatial memory span Abstract thinking, motor sequencing task, inhibitory control etc.	Digital symbol substitution test, finger tapping test Visual spatial capacity memory test Frontal assessment battery	There was a significant difference in the number of finger taps between 0 and 12 h and 6 and 12 h. There was a significant decrease in the number of correct responses in the digit symbol substitution test at 0 and 12 h of work. There was no significant difference in the visual spatial memory test and in the scores obtained in frontal assessment battery test.
Rouch et al. (2005)	France	Evaluate, on a large cross-sectional sample of workers, the long-term influence of shift-work on verbal memory and speed performances	*N* = 3,237 workers	Memory Executive functions Attention	Verbal memory Speed performances Selective attention	Adaptation of Rey verbal learning test Digit-symbol substitution subtest Test derived from Sternberg test	Cognitive functioning tends to be impaired by a long-term exposure to shift-work.
Saricaoğlu et al. (2005)	Turkey	Evaluate the attention, learning and memory related cognitive functions after 12-h day versus night shift-work in anaesthesia residents	*N* = 33 Anaesthesia residents: 15 working on day shift 18 working on night shift	Memory/learning	Short-term memory	Auditory verbal learning test, visual aural digit span test	The cognitive functions of residents may be impaired after the night shift.
Shwetha and Sudhakar (2012)	India	Study the cognitive functions in male BPO employees exposed to regular shifts	*N* = 100: 50 Business process outsourcing (BPO) shift workers 50 Non-BPO employees not working in shifts	Executive functions Memory/learning Attention	Working Memory Verbal Memory Sustained attention, selective attention	NBack Auditory verbal learning test Digit vigilance test, digital symbol substitution test, Stroop test	BPO employees performed poorly compared to their controls in tests for mental speed, learning and memory, and response inhibition. No changes were seen between groups in tests for attention and working memory. Cognitive functions are impaired in BPO employees exposed to regular shift changes.
Shwetha and Sudhakar (2014)	India	Study the cognitive functions – Learning, memory and executive function in female BPO employees exposed to regular shifts.	*N* = 100: 50 Business process outsourcing (BPO) shift workers 50 Non-BPO employees not working in shifts	Executive functions Memory/learning Attention	Verbal working memory, visual working memory Verbal memory Selective attention	Verbal and visual NBack Auditory verbal learning test Stroop test	BPO employees performed poorly compared to their controls in tests for learning and memory, response inhibition and visual working memory. No changes were seen between groups in tests for verbal working memory.
Smith et al. (1995)	United Kingdom	Study the effect of shiftwork on nuclear power workers	*N* = 22 power plant workers	Memory Attention	Associative memory Vigilance	Search and memory test Choice reaction test	Significant variations were observed in alertness and performance components during 12-h night shift.
Soares and de Almondes (2017)	Brazil	Evaluate the connection between sleep quality and visuospatial perception in 16 workers on rotating shifts from a petrochemical company	*N* = 16 petrochemical workers	Memory Attention	Visuo-spatial memory Sustained attention	Rey complex figure test Psychomotor vigilance Test	The results suggest that a good sleep quality (day shift) may be associated with better visuospatial performance.
Stout et al. (2021)	USA	Determine the impact of shift schedule on sleep duration, sleep quality, processing speed, sustained attention, vigilance, and mental health in firefighters.	*N* = 45 firefighters	Attention Executive functions	Sustained attention, divided attention Processing speed	Psychomotor vigilance task, Woodcock Johnson auditory attention, trial making test B Trial making test A	Results suggest that even minimal sleep disruption affected cognitive functioning (e.g., processing speed, visual-motor coordination, and reaction time), increasing the likelihood of poor work performance or injury.
Sun et al. (2021)	Australia	Compare cognitive function following shifts at different times of the day in an Australian Emergency Department context.	*N* = 140: 109 medical staff 31 nursing staff	Attention Executive functions	Divided attention Processing speed	Trial making test B Trial making test A	Night shift work was associated with a longer trial making test time. This may indicate a decrease in cognitive performance, visual attention, processing speed, task switching and executive function and may implicate the quality of care for patients and worker safety.
Taylor et al. (2019)	North Yorkshire, UK	Compare sleep duration, cognitive performance, and vigilance at the start and end of each shift within a three-shift, forward rotating shift pattern, common in United Kingdom police forces.	*N* = 33 police employees	Perceptual-motor function Memory/learning Attention	Motor praxis Visuo-spatial learning, working memory Sustained attention	Motor praxis task Visual object learning task, NBACK Digital symbol substitution task, psychomotor vigilance test	Results showed a significant main effect of shift type in the visual object learning task and NBACK task and also a significant main effect of start/end in the digital symbol substitution task.
Titova et al. (2016)	Sweden	Investigate whether self-reported shift work history would be associated with performance on the trail making test (TMT).	*N* = 7,143: 4,611 Non Shift-workers (SW) 1,531 Past SW 358 Recent former SW 643 Current SW	Attention Executive functions	Divided attention Processing speed	Trial making test B Trial making test A	Results indicate that shift work history is linked to poorer performance on the TMT in a cohort of middle-aged and elderly humans.
Vajravelu et al. (2016)	Puducherry, India	Evaluate cognitive functions using neurophysiological and neuropsychological methods in rotating night shift and day workers and to compare cognition between the two groups.	*N* = 80 40 male security guards (night shift at least for 6 months) 40 day workers (no night shift in last 2 years)	Attention	Vigilance, sustained attention, selective attention	Auditory reaction time and visual reaction time, digital vigilance test, Stroop test	Night-shift workers who are prone to circadian rhythm alteration will have impaired cognitive performance.
Veddeng et al. (2014)	Bergen, Norway	Assess whether gynaecologists have impaired laparoscopic skills and/or reduced cognitive function after long on-call hours.	*N* = 28 gynaecologists	Perceptual-motor functions Memory/learning Attention	Sensorimotor abilities Associative memory Vigilance	Motor screening test Paired associates learning Stocking of Cambridge and reaction time	Results showed a small increase in reaction time but no other signs of reduced cognitive function.
Williams et al. (2017)	Houston, USA	Investigate whether measurable executive function differences exist following a single overnight call versus routine shift	*N* = 60 anaesthesiology residents: 30 working daytime shifts 30 working overnight call shifts	Attention	Vigilance	Pointing task	These results indicate that overnight call residents demonstrate both sensorimotor and cognitive slowing compared to routine daytime shift residents.
Zhao et al. (2021)	China	Investigate the effects of shift work on sleep and cognitive function in the middle-aged male miners	*N* = 931 male miners: 230-night shift 701-day shift	Executive functions Memory/learning	Working memory Learning ability, immediate recall, and retention	Brief visuospatial memory test-revised Hopkins verbal learning test-revised	The workers for night shift work in adulthood would tend to have impaired working memory.

**Table 5:** Sedentary work and cognitive function: retrieved studies and their main outcomes.

Sedentary work and cognitive							
Study	Country (region)	Goal of the study	Sample	Cognitive domains investigated	Cognitive functions assessed for each domain	Cognitive tests used for each function	Synthesis of main results
Baker et al. (2018a)	Perth, Australia	Examine the impact of prolonged standings on health and productivity.	*N* = 20 workers: 13 sedentary 4 primarily standing 3 physical	Attention Executive function	Sustained attention Problem solving	Sustained attention to response test Ruff figural fluency test	Sustained attention reaction time deteriorated, while creative problem solving improved. Body discomfort was positively correlated with mental state. The observed changes suggest replacing office work sitting with standing should be done with caution.
Baker et al. (2018b)	Perth, Australia	Investigate whether use of a movement intervention when undertaking prolonged standing affected discomfort and cognitive function.	*N* = 20 workers	Attention Executive function	Sustained attention Problem solving	Sustained attention to response test Ruff figural fluency test	Standing with movement provided no advantage in discomfort or cognitive function.
Baker et al. (2018c)	Perth, Australia	Examine discomfort and two areas of cognitive function over two hours of prolonged sitting.	*N* = 20 workers	Attention Executive function	Sustained attention Problem solving	Sustained attention to response test Ruff figural fluency test	There were no substantial correlations between discomfort and cognitive function. The observed changes suggest prolonged sitting may have consequences for musculoskeletal discomfort and cognitive function and breaks to interrupt prolonged sitting are recommended.
Bojsen-Møller et al. (2019)	Sweden	Investigate associations between objectively measured physical activity and sitting behaviour, and cognitive functions.	*N* = 334 office workers	Memory/learning Executive function Attention	Episodic memory Processing speed, working memory Divided attention, selective attention	Free recall, word recognition Digit symbol substitution and trial making test A, 2-back and digit span backwords, and automated operation span Trial making test B, color Stroop task	The results indicate that the length of moderate-to-vigorous physical activity (MVPA) bouts are associated with better Stroop performance in the least fit participants, but do not support the notion that more time spent in MVPA or less time in SB is associated with better cognitive function.
John et al. (2009)	USA	Assess differences between seated and walking conditions on motor skills and cognitive function tests.	*N* = 20 workers	Attention Perceptual-motor function Executive functions	Selective attention Fine motor movement Mathematical reasoning and reading comprehension test	Stroop color and word test Computer mouse proficiency test Graduate record examination math and verbal section	Compared with the seated condition, treadmill walking caused a 6 to 11% decrease in measures of fine motor skills and math problem solving but did not affect selective attention and processing speed or reading comprehension.
Ohlinger et al. (2011)	USA	Assess participants’ ability to perform tasks requiring attention, short term memory, and simple motor skill while sitting, standing, or walking at an active workstation.	*N* = 50 workers	Memory Attention Perceptual-motor function	Immediate recall Selective attention Motor speed	Auditory consonant trigram test (ACTT) Stroop color word test (SCWT) Digital finger tapping test (DFTT)	Results indicate that adding the walking task to the ACTT and SCWT conditions results in no decrement in performance on these tasks. Conversely, adding the walking task to the DFTT condition results in reduced performance on the DFTT task.
Russell et al. (2016)	Australia	Examine the effect of sitting vs. standing for one hour per day for five consecutive days on attention, information processing speed, short-term memory, working memory and task efficiency.	*N* = 36 university staff	Attention Executive function	Selective attention, Sustained attention, divided attention Working memory, processing speed	Stroop test, four-choice visual reaction time test, trial making test B Digit span, letter number sequencing, digit symbol coding, trial making test A	The results of the study showed no statistically significant difference in cognitive performance or work efficiency between the sitting and standing conditions. This result suggests that the use of sit-stand workstations is not associated with a reduction in cognitive performance.

**Table 6:** Occupational stress and cognitive function: retrieved studies and their main outcomes.

Occupational stress and cognitive							
Study	Country (region)	Goal of the study	Sample	Cognitive domains investigated	Cognitive functions assessed for each domain	Cognitive tests used for each function	Synthesis of main results
Cano-López et al. (2023)	Valencia, Spain	Characterize the burnout level of primary healthcare professionals working in rural areas, and to analyse its relationship with executive functioning	*N* = 32 primary healthcare professionals	Executive functions Attention Language	Processing speed, cognitive flexibility Selective attention Phonemic fluency, semantic fluency	Trial making test A, Wisconsin card sorting test Stroop color-word task F, A, and S words, animals words	Results suggest that burnout in healthcare professionals could have a detrimental effect on the efficiency of health systems, and it is related to poor executive functioning.
de Souza-Talarico et al. (2020)	São Paolo, Brazil	Aim to investigate whether work-related stress is associated with low cognitive performance in middle aged adults.	*N* = 9,969 workers	Attention Language Memory	Divided attention Verbal Fluency Free recall	Trail Making Test B Verbal fluency test Delayed recall word test	Work-related stress is associated with lower performance on the delayed recall, verbal fluency, and executive function tests in middle-aged adults.
Eskildsen et al. (2015)	Denmark	Examine whether patients with work-related stress complaints have cognitive impairments compared to a matched control group without stress.	*N* = 118 workers: 59 with work-related stress complaints 59 healthy controls	Memory Executive functions Language Perceptual-motor function	Immediate and general memory, prospective memory Working memory, processing speed, calculation ability Vocabulary Visuo-motor abilities	WAIS III, prospective memory test WAIS III, PASAT Vocabulary from WAIS III Rey complex figure test	Compared to controls, patients generally showed mildly reduced performance across all the measured domains of the neuropsychological test battery. The most pronounced differences between patients and controls were seen on tests of prospective memory, speed and complex working memory.
Elovainio et al. (2009)	London, UK	Test associations between high strain and active jobs and cognitive function in middle-aged men and women.	*N* = 4,146 servants (aged 35–55 years) 2,989 men 1,157 women	Memory Executive function Language	Verbal memory Inductive reasoning Verbal meaning, phonemic and semantic fluency	Free word recall test Alice Heim intelligence test Mill Hill vocabulary test, S words and animal words	Longer exposure to high job strain was associated with lower scores in most of the cognitive performance tests. However, these associations disappeared on adjustment for employment grade. Phonemic fluency was an exception to this pattern. In these data associations between cumulative exposure to high strain and cognition are largely explained by socioeconomic position.
Farahat et al. (2022)	El Cairo, Egypt	Assess work-related stress and burnout experienced by health care workers and its impact on the cognitive domain of their executive functioning.	*N* = 81 health care workers	Executive functions	Cognitive flexibility	Wisconsin card sorting test	Health care workers on the front line experienced a high degree of work-related stress in addition to burnout in the form of emotional exhaustion, depersonalization, and reduced personal achievement. They also suffered from impaired cognitive executive functioning due to such stressful exposure.
Gafarov et al. (2021)	Novosibirsk, Russia	Investigate the effect of workplace stress on cognitive functions of young men and women.	*N* = 1,009: 463 men 546 women	Attention Memory Language	Sustained attention Immediate and delayed recall Verbal fluency	Burdon’s test A.R.Luria 10-words learning task Animal naming test	Younger adults experiencing workplace stress have a decrease in cognitive functions.
Gutshall et al. (2017)	South Florida, USA	Investigate the effect of occupational stress on working memory in police officers.	*N* = 25 police officers	Memory	Immediate and delayed recall	Rey Osterreith complex figure memory test	The results of the investigation identified a deficit in working memory in Junior, Veteran, and Senior Officers, based on the Ray Osterreith Complex Figure Scores at Baseline (pre-stress) vs. Test Day (post-stress).
Landolt et al. (2017)	Australia	Assess the relationship of work stress with indices of stress physiology and decision-making and reaction time.	*N* = 32 jockeys	Executive functions	Decision making	Chase, detection, and identification. CogState battery	Higher occupational stress was correlated with the cortisol awakening responses (high stress *r* = −0.37; low stress *r* = 0.36), and with decrements in decision-making.
Nguyen et al. (2012)	USA	Examine the relationship between stress and change in stress with change in cognitive function in Latino farmworkers	*N* = 123 farmworkers	Memory Attention	Visual memory Divided attention	Benton visual retention test Trail making test B	Stress at work is an important risk factor for poor cognitive function. This analysis suggests several implications for the provision of health care and for the organization of work for farmworkers.
Van Der Linden et al. (2005)	Netherlands	Test whether self-reported cognitive failures in burned out workers are associated with objective measures of attentional deficits.	*N* = 43 workers: 13 clinical burnout 16 high burnout nonclinical 14 no-burnout	Attention	Sustained attention	Sustained attention to response test and Burdon-Wiersma test	Results indicate that burnout is associated with difficulties in voluntary control over attention and that the level of such difficulties varies with the severity of burnout symptoms.
Vuori et al. (2014)	Finland	Examine the association between work stress and cognitive performance.	*N* = 99 women working in hospital: *N* = 43 high stress *N* = 56 low stress	Language Memory Executive functions	Verbal fluency, vocabulary Delayed recall, immediate recall Working memory, mathematics ability, processing speed	Animal words, WAIS-III vocabulary WMS-III word lists, Rey 15-item memory test WAIS III-digit span, WAIS-III arithmetic, WAIS-III letter-number sequencing	The association found between job stress and speed of memory retrieval might be one important factor explaining the effect of stress on work performance.
Zhuo et al. (2021)	European countries and Israel	Examine the association of job strain with cognitive ability and the influence of life-course job strain on later life cognitive decline.	*N* = 13,349: Active jobs = 2,262 Passive jobs = 4,407 Low strain job = 3,658 High strain job = 3,022	Memory Executive functions	Episodic memory Verbal functioning	Immediate and delayed recall test Verbal fluency test	Both passive and high strain jobs were associated with lower levels of cognitive ability (episodic memory and verbal fluency) in comparison with active job. Long exposure to active- or low strain-job was associated with higher cognitive ability whereas long exposure to passive job or moderate duration of high strain job was associated with lower cognitive ability. The rate of memory decline was positively related to moderate duration of passive job and negatively related to long-term exposure to low strain job.

**Table 7:** Prolonged working hours and cognitive function: retrieved studies and their main outcomes.

Prolonged working hours and cognitive							
Study	Country (region)	Goal of the study	Sample	Cognitive domains investigated	Cognitive functions assessed for each domain	Cognitive tests used for each function	Synthesis of main results
Ansiau et al. (2008)	Southern France	Examine the consequences of working conditions on the previous day on cognitive performance the following day.	*N* = 2,337 workers	Memory Executive function Attention	Verbal Memory Processing speed Selective attention	Adaptation of Rey verbal learning test Digital-symbol substitution test Adaptation of Sternberg test	Results showed that both physical activity and working before 6 am or after 10 pm on the previous day were significantly associated with poorer cognitive performance.
Proctor et al. (1996)	USA	Test whether increased overtime work predicts impairment in cognitive performance in the domains of attention, executive function, and mood.	*N* = 206 automotive workers: *N* = 69 who worked no overtime *N* = 137 who worked some overtime (over 8 h)	Attention Executive functions Memory Language	Divided attention, sustained attention Cognitive flexibility Short-term memory Verbal ability	Trail making test, symbol-digit substitution test Wisconsin card sorting test Visual reproduction, delayed recognition span test, pattern memory Vocabulary	Cross-sectional data analysis by multiple linear regression, after adjustment (see column “Statistics”) demonstrated that increased overtime was significantly associated with impaired performance on several tests of attention and executive function.
Virtanen et al. (2009)	London, UK	Examine the association between long working hours and cognitive function in middle age, with a 5-year follow-up.	*N* = 2,214 servants (aged 35–55 years) 1,694 men 520 women	Memory Executive function Language	Short-term memory Fluid and crystallized intelligence Phonemic fluency and verbal fluency	20-word Free recall test Alice Heim 4-I and Mill Hill vocabulary test S words and animal words	Compared with working 40 h per week at most, working more than 55 h per week was associated with lower scores in the Mill Hill vocabulary test at both baseline and follow-up. Long working hours also predicted decline in performance on the reasoning test (Alice Heim 4-I). This study shows that long working hours may have a negative effect on cognitive performance in middle age.

**Table 8:** Expertise and cognitive function: retrieved studies and their main outcomes.

Expertise and cognitive							
Study	Country (region)	Goal of the study	Sample	Cognitive domains investigated	Cognitive functions assessed for each domain	Cognitive tests used for each function	Synthesis of main results
Colonia-Willner (1998)	San Paolo, Brazil	Determine which component of intelligence is best for predicting bank managers performance.	*N* = 200 bank managers: 43 experts 157 non experts	Intelligence	Tacit knowledge Abstract reasoning Verbal reasoning	Tacit knowledge inventory for managers (TKIM) Raven’s advanced progressive matrices Verbal reasoning subtest of the differential aptitude test	TKIM time taken by participants to grade the 91 strategies was longer among nonexperts than among experts. Abstract reasoning was higher among experts than among non-experts. Verbal reasoning was significantly higher among experts than non-experts. The results suggest that stabilization of some aspects of intelligence may occur in old age (experts).
Ovaskainen and Heikkilä (2007)	Finland	Determine the cognitive abilities of cut-to-length single-grip timber harvester operators and operator students.	*N* = 46 harvester operators: 6 Professional (assessed trough WAIS, AVO and WMS) 40 Students (assessed trough AVO, WAIS-MR, DS, SS)	Memory Attention Perceptual-motor function Executive function	Long-and-short term memory Attention span Visuospatial abilities and psychomotor Concentration and non-verbal deduction	WAIS-III(PC), WAIS-III(DS), WAIS-III(SS), WMS-R WAIS-III(PC), WAIS-III(SS), WMS-R AVO-9 S2, AVO-9 S3, WAIS-III(PC), WAIS-III(BD), WAIS-III(DS), WAIS-III(SS) AVO-9 V5, WAIS-III(PC), WAIS-III(MR), WAIS-III(DS), WAIS-III(SS)	The results show comprehensive perception, wide use of memory functions, non-verbal deduction, spatial perception, coordination, concentration, and motivation are characteristics of a productive operator. The most productive operators did not possess any superior abilities and, therefore, the most important attribute appears to be a good mastering of different kinds of abilities.
Wagner-Hartl et al. (2018)	Graz, Austria	Investigate deal with possible age-related impairments of sensory functions and work performance during extended working time, between older and younger workers.	*N* = 55 white-collar workers: 32 young 23 old	Attention Executive functions	Long-term selective attention and concentration Reaction time	DAUF sustained attention S3 (DAUFS3) and COGNITRONE S2 (COGS2) Reaction-test S5 (RTS5)	The results show that older participants reacted slower than younger participants did. Furthermore, younger participants reacted more frequently in a correct way.

Study = name of authors, year; Country (Region) = where the study took place; Goal of study = Objective of the study; Sample = sample size; Cognitive domains investigated = which cognitive domain were investigated; Cognitive functions assessed for each domain = which cognitive functions were investigated for each domain; Cognitive tests used for each function = which cognitive test were used to evaluate each cognitive function; Synthesis of main results = narrative summary of results.

#### 2.2.7 Study risk of bias assessment

We assessed the risk of bias for each study in Supplementary Table S2 by compiling the AXIS tool (Downes et al., 2016).

## 3 Results

### 3.1 Flow diagram

**Figure 1:**
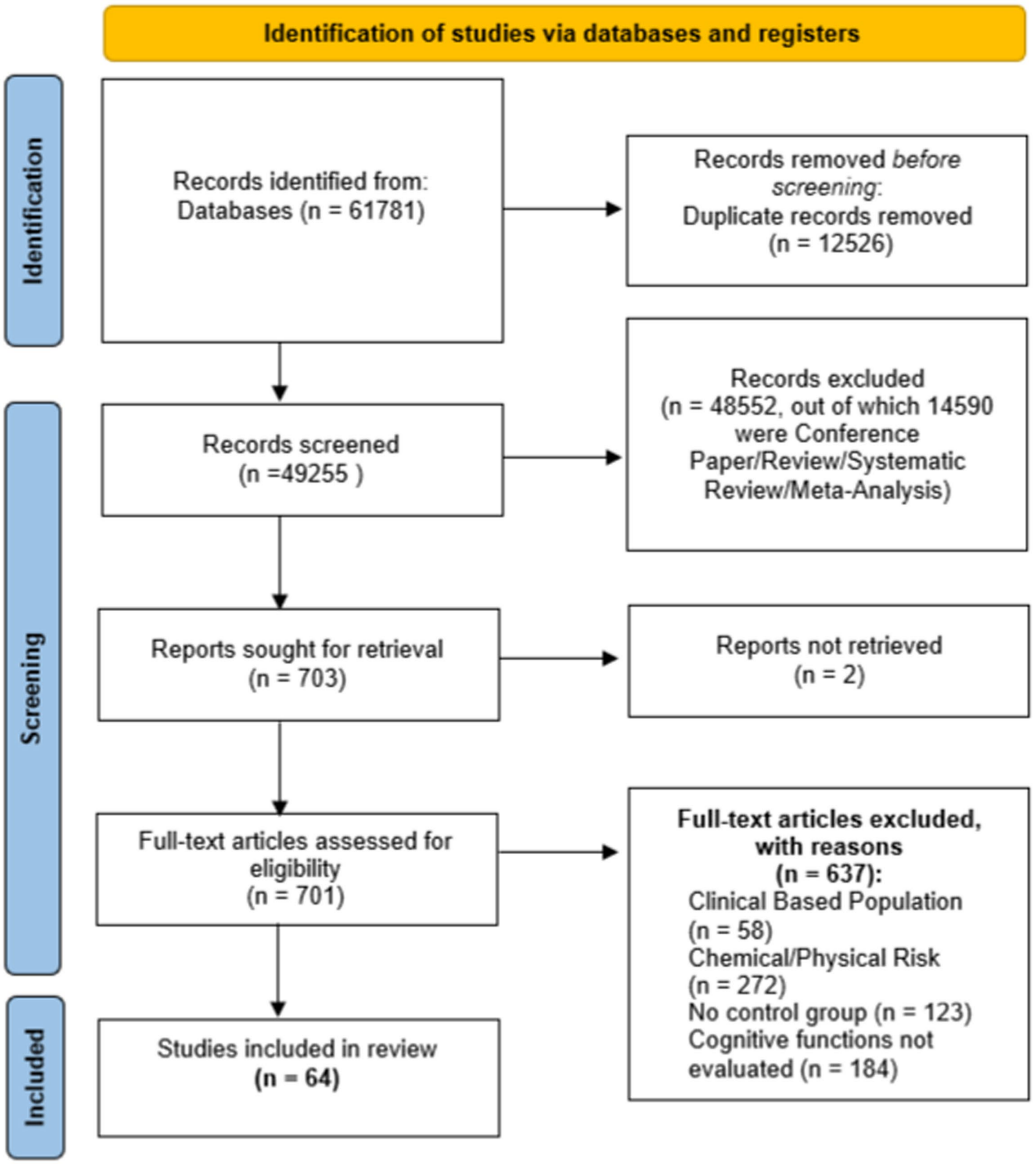
Flow diagram.

Figure 1 shows all the phases in the systematic review process. The research carried out on PubMed and Scopus yielded 13,455 and 48,066 studies, respectively. These 61,781 papers were merged in a non-redundant database and 49,255 papers remained. Then, by eliminating all studies not related to our research question and all studies that did not match PICOS criteria related to Study Design (exclusion for all types of reviews, meta-analysis, and conference papers), the number of studies was reduced to 703, of which 2 were not retrieved. Finally, by applying all the PICOS criteria, we obtained 64 studies to be included in the systematic review.

### 3.2 Study selection and characteristics

Two independent reviewers (P.B. and M.L.) checked the pool of 49,255 abstracts collected from PubMed and Scopus search engine outputs; any disagreement was discussed with M.B. as the arbiter. Titles and abstracts were screened, and 48,550 were removed through a semi-automatic script created via MATLAB (The MathWorks, Inc., Natick, MA, United States), according to PICOS criteria related to Study Design (14,590 papers) and irrelevance to the research question (34,258 papers). The remaining 703 papers were checked for eligibility according to the remaining PICOS criteria, all clinical based population studies (58), all studies regarding chemical/physical risk (272), all studies with no control group (123) and lastly, all studies without a quantitative evaluation of cognitive functions (184) were excluded, 2 papers were not retrieved even after explicit request to the corresponding authors. Finally, 64 articles were included, summarized in Tables 4–8, which shows the main information of each, divided according to work-related factors that may affect cognitive functions.

### 3.3 Synthesized findings

In this subsection, for each study, we reported the main findings of pertinence for this systematic review grouped according to work-related factors that may affect cognitive functions.

#### 3.3.1 Shift work and cognitive functions

Among 64 studies included in the systematic review, 39 were related to the effect of shift work on cognitive functions (Table 4) (Deary and Tait, 1987; Orton and Gruzelier, 1989; Lingenfelser et al., 1994; Smith et al., 1995; Petru et al., 2005; Rouch et al., 2005; Saricaoğlu et al., 2005; Griffiths et al., 2006; Chang et al., 2011, 2013a,b; Anderson et al., 2012; Shwetha and Sudhakar, 2012, 2014; Niu et al., 2013; Özdemir et al., 2013; Veddeng et al., 2014; Kazemi et al., 2016, 2018; Maltese et al., 2016; Titova et al., 2016; Vajravelu et al., 2016; Haidarimoghadam et al., 2017; Soares and de Almondes, 2017; Williams et al., 2017; Persico et al., 2018; Taylor et al., 2019; Abdelhamid et al., 2020; Adams and Venter, 2020; Athar et al., 2020; James et al., 2021; Prasad et al., 2021; Stout et al., 2021; Sun et al., 2021; Zhao et al., 2021; An et al., 2022; Benítez-Provedo et al., 2022; Esmaily et al., 2022; Peng et al., 2022).

Abdelhamid et al. (2020) evaluated the differences in cognitive functions among 50 anaesthesia residents before and after a 24-h shift and found that after the shift their cognitive processes were impaired. Adams and Venter (2020) wanted to quantify the effects of shift work on cognitive functions in anaesthesiology trainees and observed that after 14-h night shift they had a decline in reaction time, psychomotor functions and attention. An et al. (2022) explored the effects of different work shifts on cognitive and executive performance in nurses, showing that reported fatigue after the evening shift can negatively affect reaction time. Anderson et al. (2012) sought to quantify the cognitive deterioration due to repeated exposure to 24- to 30-h shift and found progressive impairment due to chronic sleep deficiency. Athar et al. (2020) investigated the influence of shift work on executive functions, finding impairment of areas deputed to executive functions, such as prefrontal area. Benítez-Provedo et al. (2022) evaluated the change in several cognitive functions after a guard shift in physicians, noting lower performance in executive functions and attention. Chang and colleagues carried out three studies (Chang et al., 2011, 2013a,b) on nurses: in the first one (2011) they compared cognitive performance at the time of maximum fatigue (3–4 a.m. on the last night shift of the rotation) among nurses who had been working for two, three, or four consecutive night shifts; the results showed greater impairment of perceptual and motor ability after two consecutive night shifts compared with four consecutive night shifts; in the second (Chang et al., 2013a), they explored changes in cognitive functions in the daytime of nurses working on fast rotating shifts and found a poorer performance on visual attentive task; in the third (Chang et al., 2013b), they explored the same changes as the previous study but after one block of fast forward rotating shift (2 days, 2 evenings, and 2 nights), finding no changes in cognitive performance. Deary and Tait (1987) evaluated the effect of sleep loss due to night shift on cognitive performance of medical house officers and found a negative effect on memory. Esmaily et al. (2022) evaluated the relationship between shift work and cognitive performance in nurses and the study showed that shift work can impair working memory and attention especially after a night shift. Griffiths et al. (2006) characterized the cognitive performance of anaesthetic registrars before and after a night shift noting a deterioration in performance speed at the end of night shift and a decline in cognitive performance after a series of night shifts. Haidarimoghadam et al. (2017) estimated the effects of consecutive night shifts and shift length on cognitive performance in a sample of room operators, finding a significant negative impact on reaction time and a reduction in correct responses and response time to the working memory task. James et al. (2021) determined the extent of changes in cognition in nurses after three consecutive 12-h shifts, detecting a significant negative effect on reaction time and number of psychomotor lapses. Kazemi and colleagues carried out two studies (Kazemi et al., 2016, 2018) on control room operators: in the first (Kazemi et al., 2016), they found a decline in cognitive performance after day and night shifts; in the second (Kazemi et al., 2018), they measured cognitive performance after seven and four consecutive night shifts and detected that those who worked seven consecutive night shifts had significantly better cognitive performance than those who worked four consecutive night shifts. Lingenfelser et al. (1994) evaluated cognitive functions in hospital doctors after a night shift, noting a considerable decrease. Maltese et al. (2016) assessed cognitive performance in a group of intensivist doctors after a night shift and found that it was significantly impaired. Niu et al. (2013) investigated selective attention in nurses with different shifts, noting that after the night shift both attention and operation speed of work were impaired. Orton and Gruzelier (1989) studied the effects of night shifts on young doctors and concluded that working long hours may adversely affect the cognitive functions. Özdemir et al. (2013) analysed the effects of shift work on health care workers’ memory, attention, and learning, finding a significantly poorer cognitive performance after night shift, particularly for working memory. Peng et al. (2022) noted a decrease in decision making competence due to night shift among nurses. Persico et al. (2018) evaluated cognitive performance of emergency physician after a night shift of 14 h or 24 h compared with a rest night at home and found a significant alteration of cognitive abilities after a 24-h shift but not after a 14-h shift. Petru et al. (2005) compared cognitive and psychomotor performance between night shift and early-late shift in a sample of automobile workers; the results indicated that, if chosen voluntarily, night shift had no immediate effects on performance compared with early-late shift. Prasad et al. (2021) evaluated changes in cognitive functions after 6 or 12 h shift compared with a day off in a sample of anaesthesia residents; they found significant differences in sustained attention performance between the 6 and 12 shift and the day off but not for memory and executive functions. Rouch et al. (2005) studied the long-term influence of shift work on verbal memory and speed performances, demonstrating a trend toward impairment over the long term. Saricaoğlu et al. (2005) investigated memory and learning between 12-h day shift and night shift in a sample of anaesthesia residents, noting that cognitive functions may be impaired after the night shift. Shwetha and Sudhakar (2012, 2014) carried out two studies: in both studies they compared cognitive functions in business process outsourcing (BPO) shift workers versus non-BPO employees not working in shifts; it was found that BPO employees performed poorly in mental speed, learning, memory, and response inhibition but not in working memory. Smith et al. (1995) studied the effect of shift work on cognitive performance in nuclear power workers, finding significant variations in alertness and performance during 12-h night shift. Soares and de Almondes (2017) explored the connection between sleep quality (night shift versus day shift) and visuospatial perception in petrochemical workers; the results suggested that good sleep quality (day shift) could be associated with better visuospatial performance. Stout et al. (2021) sought to determine the impact of shift work on cognitive functions in firefighters and reported that even minimal sleep disruption, caused by night shift, affected cognitive functioning, increasing risk of poor work performance or injury. Sun et al. (2021) compared cognitive functions among different work shifts in an emergency department and found night shift work to be associated with a decrease in cognitive performance. Taylor et al. (2019) compared cognitive performance and vigilance at the start and end of each work shift within a three-shift schedule in police forces; the results showed a significant main effect of shift type in visuo-spatial task. Titova et al. (2016) investigated whether self-reported shift work history had an association with cognitive performance and results indicated that shift work history was related to poorer performance. Vajravelu et al. (2016) compared cognitive functions in night shift workers and day workers finding an alteration of attention in night shift workers. Veddeng et al. (2014) assessed whether gynaecologists have impaired laparoscopic skills and/or reduced cognitive functions after long on-call hours; results showed a small increase in reaction time but no other signs of reduced cognitive functions. Williams et al. (2017) investigated whether measurable executive function differences existed following a single overnight call versus routine shift, demonstrating that overnight call residents had both sensorimotor and cognitive slowing compared to routine daytime shift residents. Zhao et al. (2021) explored the effects of shift work on sleep and cognitive functions in middle-aged male miners, noting that night shift workers in adulthood would tend to have impaired working memory (Chang et al., 2013b).

#### 3.3.2 Sedentary work and cognitive functions

Among 64 studies included in the systematic review, 7 were related to the effect of sedentary work on cognitive functions (Table 5) (John et al., 2009; Ohlinger et al., 2011; Russell et al., 2016; Baker et al., 2018a,b,c; Bojsen-Møller et al., 2019).

Baker and colleagues carried out three studies (Baker et al., 2018a,b,c): in the first (Baker et al., 2018a), they examined the impact of prolonged standings on cognitive functions, finding that sustained attention deteriorated, while creative problem solving improved; in the second (Baker et al., 2018b), they investigated whether the use of a movement intervention when undertaking prolonged standing affected discomfort and cognitive functions and found no advantage in discomfort or cognitive functions; in the third (Baker et al., 2018c), they assessed cognitive functioning over 2 h of prolonged sitting and observed that increased discomfort in all areas of the body that can affect cognitive functioning. Bojsen-Møller et al. (2019) explored the associations between physical activity, sitting behaviour (SB) and cognitive functions: they found that duration of moderate-to-vigorous physical activity (MVPA) was associated with better selective attention performance but results did not support the hypothesis that more time spent in MVPA or less time in SB was associated with better cognitive functions. John et al. (2009) assessed the differences between treadmill walking and sitting conditions on motor skills and cognitive functioning during work; results showed that treadmill walking caused a decrease in fine motor skills and math problem solving compared with the sitting condition however no differences were found concerning selective attention, processing speed or reading comprehension. Ohlinger et al. (2011) estimated the ability of performing cognitive tasks in workers while sitting, standing or walking at an active workstation; results indicated that the addition of the walking task did not alter performance in immediate recall and selective attention, but caused a decline in motor speed performance. Russell et al. (2016) examined the effect of sitting versus standing for 1 h per day for five consecutive days on cognitive functions and found no differences between the two conditions.

#### 3.3.3 Occupational stress and cognitive functions

Among 64 studies included in the systematic review, 12 were related to the effect of occupational stress on cognitive functions (Table 6) (Van Der Linden et al., 2005; Elovainio et al., 2009; Nguyen et al., 2012; Vuori et al., 2014; Eskildsen et al., 2015; Gutshall et al., 2017; Landolt et al., 2017; de Souza-Talarico et al., 2020; Gafarov et al., 2021; Zhuo et al., 2021; Farahat et al., 2022; Cano-López et al., 2023).

Cano-López et al. (2023) characterized the level of burnout among primary healthcare professionals and its relationship with cognitive functions; results suggested that burnout was related to poor cognitive functioning. de Souza-Talarico et al. (2020) found that work-related stress was associated with lower performance in delayed recall, verbal fluency, and executive functions in middle aged workers. Eskildsen et al. (2015) examined whether patients presenting work-related stress complaints displayed cognitive impairment. When compared to stress-free, patients showed slightly reduced performance across all measured domains of the neuropsychological test battery. Elovainio et al. (2009) tested the association between high strain, active jobs and cognitive function in middle aged men and women; results showed that longer exposure to high strain job was associated with lower scores in most of the cognitive performance test, but these associations disappeared on adjustment for employment grade. Farahat et al. (2022) assessed work-related stress in healthcare workers and its impact on cognitive functions, noting an impairment due to stress exposure. Gafarov et al. (2021) investigated the effect of workplace stress on cognitive functions of young workers, and those who experienced stress displayed a decrease in cognitive functions. Gutshall et al. (2017) examined the effect of occupational stress on working memory in police officers, finding a deficit after a stressful event. Landolt et al. (2017) assessed the relationship between work stress, decision-making and reaction times in jockeys; higher occupational stress was significantly correlated with decrements in decision-making. Nguyen et al. (2012) examined the relationship between stress and change in cognitive functioning in farmworkers ascertaining that stress was an important risk factor for poor cognitive functioning. Van Der Linden et al. (2005) evaluated the association between burnout levels and attentional deficits, finding that burnout was associated with difficulties in voluntary control over attention and that the level of such difficulties varied along with the severity of burnout symptoms. Vuori et al. (2014) investigated the relationship between work stress and cognitive performance, noting a negative association between work stress and speed of memory retrieval. Zhuo et al. (2021) evaluated the association of job strain with cognitive ability and finding that high strain jobs were associated with lower levels of cognitive ability.

#### 3.3.4 Prolonged working hours and cognitive functions

Among 64 studies included in the systematic review, 3 were related to the effect of prolonged working hours on cognitive functions (Table 7) (Proctor et al., 1996; Ansiau et al., 2008; Virtanen et al., 2009).

Ansiau et al. (2008) examined the consequences of working condition on the previous day on cognitive performance the following day, finding that both physical activity and working before 6 a.m. or after 10 p.m. on the previous day were significantly associated with poorer cognitive performance. Proctor et al. (1996) evaluated whether increased overtime work predicted impairment in cognitive performance, finding that increased overtime was significantly associated with impaired performance on several tests of attention and executive function. Virtanen et al. (2009) examined the association between long working hours and cognitive function in middle age and results showed that long working hours may have a negative effect on cognitive performance in middle age.

#### 3.3.5 Expertise and cognitive functions

Among 64 studies included in the systematic review, 3 were related to the effect of expertise on cognitive functions (Table 8) (Colonia-Willner, 1998; Ovaskainen and Heikkilä, 2007; Wagner-Hartl et al., 2018).

Colonia-Willner (1998) sought to determine differences in cognitive performance in the domain of intelligence between expert and non-expert bank managers, and the results suggested that stabilization of some aspects of intelligence may occur in older age when workers are more experienced. Ovaskainen and Heikkilä (2007) determined the differences in cognitive abilities and productivity between timber harvester operators and students operators; results showed no differences in cognitive abilities but the highest productivity level among students was almost as the lowest productivity of the operators. Wagner-Hartl et al. (2018) examined cognitive functions in young and old (expert) white-collars during extended working time, finding slower reaction time and more error in older workers.

## 4 Discussion

Building on the three main theories “*Use-it-or-lose-it”* (Hultsch et al., 1999; Salthouse, 2006, 2016), “*the Brain or Cognitive reserve hypothesis”* (Mortimer and Graves, 1993; Stern, 2002; Fratiglioni and Wang, 2007) and, *“Schooler’s theory of environmental influences on cognitive functioning”* (Schooler, 1984, 1990) which describe how different kinds of job and different work environments can influence the cognitive aging in a normative way, the question we asked was: “What happens to this normative functioning when factors intrinsic to the work itself, or factors caused by the work, intrude in workers’ lives and impair their cognitive functioning? “. We were sure that shift work, based on the very extensive literature on the subject, caused sleep disturbances, thus dysregulation of circadian rhythm and many other alterations (Kantermann et al., 2012) that targeted cognitive functioning (Ma et al., 2020). Just as we were certain that stress, including stress in the workplace, increased burnout risk and resulted in cognitive impairment (Lupien et al., 2009) and that sedentary behaviours, on the other hand, had effects on cognitive functioning (Gafni et al., 2021), but there were still doubts about their actual impact (Magnon et al., 2018). But we also had the doubt that these were not the only factors that could influence cognitive functioning, for this reason we decided to draft a systematic review to classify the effects of different work-related factors on cognitive functions.

This review had to include studies that quantitatively assessed workers’ cognitive functions according to different work-related factors, aiming to identify potential variations in cognitive functioning between workers exposed and not exposed to these factors, as well as differences among workers with varying levels of exposure (e.g., the same workers exposed to both night shift and day shift).

At the end of the review process, we classified the included studies according to the strict criteria into five categories: “Shift Work,” “Occupational Stress,” Sedentary Work,” “Prolonged Working Hours,” and “Expertise. Therefore, two new categories (Extended Working Hours and Competence) were introduced in addition to the three assumed during the formulation of the research question.

The category with the largest number of included studies, as expected, was “shift work” (39 studies out of 64): since it is well known the neurocognitive impairment it can cause (Vlasak et al., 2022), particularly when the shift is performed at night (Leso et al., 2021), and therefore many studies dealt with it as a better quantification of the possible damage is essential. The other most studied category is “occupational stress” (12 studies), followed by “sedentary work” (7 studies) and “prolonged working hours” (3 studies), while the last 3 studies are focused on the effect of “expertise” on cognitive functions.

The included studies were conducted in developed countries across all continents (North America, South America, Europe, and Asia), while studies conducted in developing countries are still lacking.

In the following paragraphs we will discuss the results for each category one by one in separate sections.

### 4.1 Principal outcomes

#### 4.1.1 Shift work and cognitive functions

Many studies focused their attention on the negative effects of shift work on circadian rhythm (Schettini et al., 2023) and consequently on sleep and cognitive functions. Particularly, most studies highlighted detrimental effects: such among healthcare workers, medical professionals, as anaesthesiologists had a decline in reaction time, problem-solving, attention, and mental flexibility after night shift (Saricaoğlu et al., 2005; Griffiths et al., 2006; Williams et al., 2017; Abdelhamid et al., 2020; Adams and Venter, 2020; Prasad et al., 2021), nurses had affected reaction time, motor ability, attention, cognitive flexibility, working memory, and decision making after work shift (Chang et al., 2011, 2013a,b; Niu et al., 2013; James et al., 2021; Sun et al., 2021; An et al., 2022; Esmaily et al., 2022; Peng et al., 2022), other categories of physicians(intensivists, emergency doctors, gynaecologists, junior doctors, hospital doctors, medical house officers, postgraduate year one physicians) had a deficit in attention, executive function, memory, motor function, and reaction time after night shift (Deary and Tait, 1987; Orton and Gruzelier, 1989; Lingenfelser et al., 1994; Anderson et al., 2012; Özdemir et al., 2013; Veddeng et al., 2014; Maltese et al., 2016; Persico et al., 2018; Benítez-Provedo et al., 2022). Other workers, such as security staff (Vajravelu et al., 2016; Athar et al., 2020), control room operators (Kazemi et al., 2016, 2018; Haidarimoghadam et al., 2017), business process outsourcing employees (Shwetha and Sudhakar, 2012, 2014), power plant workers (Smith et al., 1995), petrochemical workers (Soares and de Almondes, 2017), firefighters (Stout et al., 2021), police employees (Taylor et al., 2019), miners (Zhao et al., 2021), and generic groups of workers (Rouch et al., 2005; Titova et al., 2016) had an impairment in one or more cognitive functions. The only study reporting a slight difference result from the others was the one involving automotive workers (Petru et al., 2005) which suggest no immediate negative effects in cognitive and psychomotor performance of night shift workers when compared with other workers, but this effect is observed only when the night shift was voluntary. It is therefore clear from all these studies that shift work, particularly night work with the aggravation of altered circadian rhythms and sleep patterns, causes damage to cognitive functions, particularly those related to alertness, sustained attention and working memory, corroborating previous studies on sleep deprivation (Nilsson et al., 2005; Joo et al., 2012). These results are in line with what we expected; the immense literature attesting to the damage to cognitive functioning caused by sleep problems is corroborated by studies involving those types of work that cause dysregulation. Long-term damage may be mitigated in jobs such as physician, which are less monotonous and repetitive than an assembly line in a factory, as asserted by “*the Brain or Cognitive reserve hypothesis”* (Mortimer and Graves, 1993; Stern, 2002; Fratiglioni and Wang, 2007), but the short-term effects remain equally hazardous to health.

#### 4.1.2 Sedentary work and cognitive functions

Having sedentary behaviour more than 75% of the time was associated with cognitive impairment in middle age (Gafni et al., 2021), however the included studies have different outcomes. Some studies found no correlation between sedentary/standing behaviours and decrease in cognitive functions (Russell et al., 2016; Baker et al., 2018c; Bojsen-Møller et al., 2019); other studies tried to use movement interventions or treadmill walking to counteract sedentary behaviours but, one of these (Baker et al., 2018b) found no advantage on cognitive functions and the other two of these (John et al., 2009; Ohlinger et al., 2011) even found decreased performance in some tasks and no change in others; only one of the studies (Baker et al., 2018a) found a deterioration in sustained attention and reaction time, confirming results of previous studies. The overall results are not conclusive, partly due to the profound heterogeneity in the methods of the studies, which makes them difficult to compare even with the existing literature. Also for this category, as for the previous one, the results of the systematic review confirm the premises, the picture is still not entirely clear regarding the effect sedentary work can have on cognitive health. Sedentary work, combined with a incorrect diet and lack of physical activity, can certainly cause various health problems, such as cardiovascular disorders (Elagizi et al., 2020), which can result in cognitive impairment (Picano et al., 2014), but its effect *per se* is still not completely clear; more in-depth studies with different designs capable of isolating the sedentary factor and studying it individually, without the influence of other factors causing bias, will be necessary.

#### 4.1.3 Occupational stress and cognitive functions

Stress exposure had a high detrimental effect on cognitive performance (Chen et al., 2022) and accelerated cognitive decline (Aggarwal et al., 2014). Health care workers are generally under great pressure that puts them at high risk of burnout, which has a critical effect on executive functioning (Cano-López et al., 2023), especially when they are on the front-line, such as during COVID-19 pandemic (Farahat et al., 2022). Among hospital workers, memory can be impaired by job stress as well (Vuori et al., 2014). However, not only health care workers are affected by cognitive impairment as a result of stress, even police officers (Gutshall et al., 2017), jockeys (Landolt et al., 2017), and farmworkers (Nguyen et al., 2012). Other studies have found lower cognitive performance related to job stress in young (Gafarov et al., 2021), middle-aged (Elovainio et al., 2009; de Souza-Talarico et al., 2020) workers, and after retirement (Zhuo et al., 2021) explaining that each age group may be affected, although in different ways. In this case, the included studies have very consistent results, all indicating a relationship between stress, burnout and poor cognitive functioning. This category is very consistent with the initial hypothesis we posited: the negative effects of stress can be very insidious and affect any area of health, especially cognitive functioning, which deteriorates with lack of concentration, a sense of hopelessness and then often results or overlaps with much more serious psychiatric disorders such as depression or anxiety disorders (Golonka et al., 2019).

#### 4.1.4 Prolonged working hours and cognitive functions

Prolonged working hours category can be considered in the same way as shift work, but since it does not fit completely into the category, it deserved a separate section because while shift work has precise rules and defined hours, prolonged working hours consists precisely of working long hours without this control, without these rules, to the extent that work pervades almost the totality of a person’s daily routine. This resemblance suggests similar effects on cognitive functions: working after a certain time thereby extending one’s working hours (Ansiau et al., 2008), increasing overtime commitments (Proctor et al., 1996), and work more than 55 h per week compared with to 40 h per week (Virtanen et al., 2009), leads to a poorer cognitive performance in executive functions and attention, and could even have negative effect on cognitive performance in middle age. As expected, all these studies point in the same direction, which is that increased work hours lead to a deterioration of cognitive function that can also be expressed in middle age. In our initial hypothesis, we had not considered this category, partly because, as mentioned, it could be assimilated with shift work, but the three studies included deserved a separate category as they expressed different concepts and looked at cognitive impairment that is not caused by circadian dysregulation leading to sleep problems, but rather by an immersion in work that causes workers to pervade their daily lives.

#### 4.1.5 Expertise and cognitive functions

The studies in this category matched our inclusion criteria, but deserve special mention because they deal with how experience can modify certain cognitive functions both positively and negatively. The three included studies regarding expertise found: a better cognitive functioning on the domain of intelligence in experts compared to non-experts bank managers (Colonia-Willner, 1998), no difference related to experience but only to good mastering of different abilities in timber harvester operators (Ovaskainen and Heikkilä, 2007), and slower reaction time in older but more experienced white-collar workers than in younger workers (Wagner-Hartl et al., 2018). This category is very heterogeneous and it cannot be said whether the experience has a positive or negative effect in general, in the intellectual part of the job certainly the effect can be described as positive but in the physical part the same does not apply, so we can say that the effects vary especially depending on the type of work, so a unified result cannot be obtained.

### 4.2 Limitations

This systematic review has some limitations. First, the inclusion of keywords such as “work,” however indispensable, massively enlarged the initial pool (on the order of one million papers), forcing us to eliminate less relevant keywords so as not to make it impossible to complete the selection work; conversely, we may have missed some papers to be included. Notwithstanding this, we believe that the initial pool of 61,781 papers constituted a solid starting point for reviewing all the literature regarding the topic. Another limitation concerned the choice of PICOS criteria; we were forced to narrow the field by excluding all work concerning the assessment of cognitive functions in workers exposed to chemicals/or neurotoxic substances, or physical hazard. This choice was made to focus our effort only on work-related risk factors modifiable through workplace health and wellbeing promotion strategies, otherwise the work would have been too dispersed; unfortunately, several interesting papers that could provide information on the effects of these types of jobs on cognitive functions were excluded. The last limitation concerns the included studies, some of which have small sample size but, in some cases these sample size was justified by power analyses, in other cases this lack of reliability is pointed out in AXIS Tool (Supplementary Table S2).

## 5 Conclusion

In conclusion, our systematic review showed that shift work and occupational stress have a consistent detrimental effect on cognitive functions, which is justified by the large number of studied included that confirm the literature on the topic. Shift work leads to chronic sleep deficiency, which in the long-term causes problems with attention and working memory, especially in the category of healthcare workers (e.g., nurses) who are exposed to shifts at any age and for long periods of time. Work-related stress increases the risk of burnout, which is related to poor cognitive functioning, and this risk cuts across different jobs.

The category of prolonged working hours also shows a strong negative effect on cognitive functions, but only three studies were included, that have very large sample sizes, not sufficient to give this result the same consistency as the two in the previous categories, although the negative effects are very clear; in fact, it can have immediate effects on cognitive functions, but the worst-case scenario is when the effects manifest themselves with early cognitive impairment in middle age.

So, we found evidence that relates to our research question posed as the goal of this paper the work-related factors that most and most negatively affect cognitive function are: shift work (especially night shift), occupational stress, and, most likely although further evidence is needed, prolonged working hours. These findings suggest that workplace health and wellbeing promotion should consider, for example, reducing or rescheduling night shifts to lessen circadian disruption and inadequate sleep quality (Garde et al., 2020) and/or reducing occupational stress by creating less demanding and more resourceful work environments (Bakker and Demerouti, 2014). Another idea, supported by literature, especially during prolonged working hours, but also in the other two harmful categories, is to take micro-breaks as possible cognitive recovery mechanisms in the workplace; specifically, these are not cognitive activities such as reading short magazine articles, doing personal projects or surfing the Internet, which exacerbate the effects of the workload, but rather moments of relaxation such as short naps, walks around the office, stretching, listening to music and daydreaming, or micro-social break activities, which refer to relatively short online or offline interactions with colleagues and other people outside work while at work (Kim et al., 2017).

All these suggestions emphasise the importance of protecting the health of during the working period in order to lead them to a safer working age that can lead to a reduced risk of early cognitive decline in post-retirement life.

In contrast, results related to sedentary work and expertise have been inconclusive and extremely miscellaneous, thus more studies are needed; but on this point it is very important to emphasise, especially for studies on sedentary work, the lack of a common methodological design. These studies would benefit greatly from a group-based design, in which conditions (e.g., sedentary work habits) are isolated and compared in terms of between-subject comparisons (Schumann et al., 2022).

Finally, in order to get a total picture on the impact of work-related factors on cognitive functioning, two future prospective could be: in future systematic reviews include categories excluded *a priori* from this research (e.g., workers exposed to chemicals/or neurotoxic substances, or physical hazard), and conduct studies with larger samples (Bonzini et al., 2023) and more consistent methodologies.

## Data availability statement

The original contributions presented in the study are included in the article/Supplementary material, further inquiries can be directed to the corresponding author.

## Author contributions

PB: Conceptualization, Data curation, Investigation, Methodology, Software, Writing – original draft, Writing – review & editing. CT: Funding acquisition, Writing – review & editing. AF: Writing – review & editing. TB: Writing – review & editing. AC: Writing – review & editing. CC: Investigation, Writing – review & editing. LF: Writing – review & editing. FM: Writing – review & editing. ML: Conceptualization, Investigation, Methodology, Software, Supervision, Writing – review & editing. MB: Conceptualization, Methodology, Project administration, Supervision, Writing – review & editing.
