# Supplementary material for: The effects of work on cognitive functions: a systematic review
Source: Front Psychol. 2024 May 9;15:1351625. doi: 10.3389/fpsyg.2024.1351625 (PMC11112082; doi:10.3389/fpsyg.2024.1351625)
Supplement: Supplementary file 2 [file Table_2.docx]

| **The Appraisal tool for Cross-Sectional Studies (AXIS tool, 2016) Part 1** | | | | | | | | |
| --- | --- | --- | --- | --- | --- | --- | --- | --- |
| Study | **Abdelhamid et al., 2020** | **Adams and Venter, 2020** | **An et al., 2022** | **Anderson et al., 2012** | **Ansiau et al., 2008** | **Athar et al., 2020** | **Baker et al., 2018a** | **Baker et al., 2018b** |
| Introduction |  |  |  |  |  |  |  |  |
| 1. Were the aims/objectives of the study clear? | YES | YES | YES | YES | YES | YES | YES | YES |
| Methods |  |  |  |  |  |  |  |  |
| 2. Was the study design appropriate for the stated aim(s)? | YES | YES | YES | YES | YES | YES | YES | YES |
| 3. Was the sample size justified? | YES | NO | NO | NO | NO | NO | NO | NO |
| 4. Was the target/reference population clearly defined? (Is it clear who the research was about?) | YES | YES | YES | YES | YES | YES | YES | YES |
| 5. Was the sample frame taken from an appropriate population base so that it closely represented the target/reference population under investigation? | NO | NO | NO | YES | YES | YES | YES | NO |
| 6. Was the selection process likely to select subjects/participants that were representative of the target/reference population under investigation? | YES | YES | YES | YES | YES | YES | YES | YES |
| 7. Were measures undertaken to address and categorise non-responders? | NO | NO | NO | YES | NO | NO | NO | NO |
| 8. Were the risk factor and outcome variables measured appropriate to the aims of the study? | YES | YES | YES | YES | YES | YES | NO | YES |
| 9. Were the risk factor and outcome variables measured correctly using instruments/ measurements that had been trialled, piloted or published previously? | YES | YES | YES | YES | YES | YES | NO | YES |
| 10. Is it clear what was used to determined statistical significance and/or precision estimates? (eg, p values, CIs) | YES | YES | YES | YES | YES | YES | YESY | YES |
| 11. Were the methods (including statistical methods) sufficiently described to enable them to be repeated? | YES | YES | YES | YES | NO | YES | YES | YES |
| Results |  |  |  |  |  |  |  |  |
| 12. Were the basic data adequately described? | YES | YES | YES | YES | YES | YES | YES | YES |
| 13. Does the response rate raise concerns about non-response bias? | NO | NO | NO | NO | NO | NO | NO | NO |
| 14. If appropriate, was information about non-responders described? | NON-APPROPRIATE | NON-APPROPRIATE | NON-APPROPRIATE | YES | NON-APPROPRIATE | NON-APPROPRIATE | NON-APPROPRIATE | NON-APPROPRIATE |
| 15. Were the results internally consistent? | YES | YES | NO | YES | YES | YES | YES | YES |
| 16. Were the results for the analyses described in the methods, presented? | YES | YES | YES | YES | YES | YES | YES | YES |

| **The Appraisal tool for Cross-Sectional Studies (AXIS tool, 2016) Part 2** | | | | | | | | |
| --- | --- | --- | --- | --- | --- | --- | --- | --- |
| Study | **Baker et al., 2018c** | **Benítez-Provedo et al., 2022** | **Bojsen-Møller et al., 2019** | **Cano-López et al., 2023** | **Chang et al., 2013a** | **Chang et al., 2013b** | **Chang et al., 2011** | **Colonia-Willner, 1998** |
| Introduction |  |  |  |  |  |  |  |  |
| 1. Were the aims/objectives of the study clear? | YES | YES | YES | YES | YES | YES | YES | YES |
| Methods |  |  |  |  |  |  |  |  |
| 2. Was the study design appropriate for the stated aim(s)? | YES | YES | YES | YES | YES | YES | YES | YES |
| 3. Was the sample size justified? | NO | YES | NO | NO | NO | NO | NO | NO |
| 4. Was the target/reference population clearly defined? (Is it clear who the research was about?) | NO | YES | YES | YES | YES | YES | YES | YES |
| 5. Was the sample frame taken from an appropriate population base so that it closely represented the target/reference population under investigation? | NO | YES | YES | YES | YES | YES | YES | YES |
| 6. Was the selection process likely to select subjects/participants that were representative of the target/reference population under investigation? | YES | YES | YES | YES | YES | YES | YES | YES |
| 7. Were measures undertaken to address and categorise non-responders? | NO | NO | YES | NO | NO | NO | NO | NO |
| 8. Were the risk factor and outcome variables measured appropriate to the aims of the study? | YES | YES | YES | YES | NO | YES | YES | YES |
| 9. Were the risk factor and outcome variables measured correctly using instruments/ measurements that had been trialled, piloted or published previously? | YES | YES | YES | YES | NO | YES | YES | YES |
| 10. Is it clear what was used to determined statistical significance and/or precision estimates? (eg, p values, CIs) | YES | YES | YES | YES | YES | YES | NO | YES |
| 11. Were the methods (including statistical methods) sufficiently described to enable them to be repeated? | YES | YES | YES | YES | YES | YES | NO | YES |
| Results |  |  |  |  |  |  |  |  |
| 12. Were the basic data adequately described? | YES | YES | YES | YES | YES | YES | YES | YES |
| 13. Does the response rate raise concerns about non-response bias? | NO | NO | NO | NO | NO | NO | NO | NO |
| 14. If appropriate, was information about non-responders described? | NON-APPROPRIATE | NON-APPROPRIATE | YES | NON-APPROPRIATE | NON-APPROPRIATE | NON-APPROPRIATE | NON-APPROPRIATE | NON-APPROPRIATE |
| 15. Were the results internally consistent? | NO | YES | YES | YES | YES | YES | YES | YES |
| 16. Were the results for the analyses described in the methods, presented? | YES | YES | YES | YES | YES | YES | YES | YES |

| Study | **de Souza-Talarico et al., 2020** | **Deary and Tait, 1987** | **Elovainio et al., 2009** | **Eskildsen et al., 2015** | **Esmaily et al., 2022** | **Farahat et al., 2022** | **Gafarov et al., 2021** |
| --- | --- | --- | --- | --- | --- | --- | --- |
| Introduction |  |  |  |  |  |  |  |
| 1. Were the aims/objectives of the study clear? | YES | YES | YES | YES | YES | YES | YES |
| Methods |  |  |  |  |  |  |  |
| 2. Was the study design appropriate for the stated aim(s)? | YES | YES | YES | YES | YES | YES | YES |
| 3. Was the sample size justified? | NO | NO | YES | YES | NO | YES | NO |
| 4. Was the target/reference population clearly defined? (Is it clear who the research was about?) | YES | YES | YES | YES | YES | YES | YES |
| 5. Was the sample frame taken from an appropriate population base so that it closely represented the target/reference population under investigation? | YES | NO | YES | YES | NO | YES | YES |
| 6. Was the selection process likely to select subjects/participants that were representative of the target/reference population under investigation? | YES | YES | YES | YES | YES | YES | YES |
| 7. Were measures undertaken to address and categorise non-responders? | NO | NO | NO | YES | NO | YES | YES |
| 8. Were the risk factor and outcome variables measured appropriate to the aims of the study? | YES | NO | YES | YES | YES | YES | YES |
| 9. Were the risk factor and outcome variables measured correctly using instruments/ measurements that had been trialled, piloted or published previously? | YES | NO | YES | YES | YES | YES | YES |
| 10. Is it clear what was used to determined statistical significance and/or precision estimates? (eg, p values, CIs) | YES | YES | YES | YES | YES | YES | YES |
| 11. Were the methods (including statistical methods) sufficiently described to enable them to be repeated? | YES | YES | YES | YES | YES | YES | YES |
| Results |  |  |  |  |  |  |  |
| 12. Were the basic data adequately described? | YES | YES | YES | YES | YES | YES | YES |
| 13. Does the response rate raise concerns about non-response bias? | NO | NO | NO | NO | NO | NO | NO |
| 14. If appropriate, was information about non-responders described? | NOT-APPROPRIATE | NOT-APPROPRIATE | NOT-APPROPRIATE | NOT-APPROPRIATE | NOT-APPROPRIATE | NOT-APPROPRIATE | NOT-APPROPRIATE |
| 15. Were the results internally consistent? | YES | YES | YES | YES | YES | YES | YES |
| 16. Were the results for the analyses described in the methods, presented? | YES | YES | YES | YES | YES | YES | YES |

| **The Appraisal tool for Cross-Sectional Studies (AXIS tool, 2016) Part 4** | | | | | | | | |
| --- | --- | --- | --- | --- | --- | --- | --- | --- |
| Study | **Griffiths et al., 2006** | **Gutshall et al., 2017** | **Haidarimoghadam et al., 2017** | **James et al., 2021** | **John et al., 2009** | **Kazemi et al., 2016** | **Kazemi et al., 2018** | **Landolt et al., 2017** |
| Introduction |  |  |  |  |  |  |  |  |
| 1. Were the aims/objectives of the study clear? | YES | YES | YES | YES | YES | YES | YES | YES |
| Methods |  |  |  |  |  |  |  |  |
| 2. Was the study design appropriate for the stated aim(s)? | YES | YES | YES | YES | YES | YES | YES | YES |
| 3. Was the sample size justified? | NO | NO | NO | YES | NO | NO | NO | NO |
| 4. Was the target/reference population clearly defined? (Is it clear who the research was about?) | YES | YES | YES | YES | YES | YES | YES | YES |
| 5. Was the sample frame taken from an appropriate population base so that it closely represented the target/reference population under investigation? | NO | YES | YES | NO | NO | YES | YES | YES |
| 6. Was the selection process likely to select subjects/participants that were representative of the target/reference population under investigation? | YES | YES | YES | YES | YES | YES | YES | YES |
| 7. Were measures undertaken to address and categorise non-responders? | NO | YES | NO | NO | NO | NO | NO | NO |
| 8. Were the risk factor and outcome variables measured appropriate to the aims of the study? | NO | YES | YES | YES | YES | NO | NO | YES |
| 9. Were the risk factor and outcome variables measured correctly using instruments/ measurements that had been trialled, piloted or published previously? | NO | YES | YES | YES | YES | NO | NO | YES |
| 10. Is it clear what was used to determined statistical significance and/or precision estimates? (eg, p values, CIs) | YES | YES | YES | YES | YES | YES | YES | YES |
| 11. Were the methods (including statistical methods) sufficiently described to enable them to be repeated? | YES | YES | YES | YES | YES | YES | YES | YES |
| Results |  |  |  |  |  |  |  |  |
| 12. Were the basic data adequately described? | YES | YES | YES | YES | YES | YES | YES | YES |
| 13. Does the response rate raise concerns about non-response bias? | NO | NO | NO | NO | NO | NO | NO | NO |
| 14. If appropriate, was information about non-responders described? | NOT-APPROPRIATE | NO | NOT-APPROPRIATE | NOT-APPROPRIATE | NOT-APPROPRIATE | NOT-APPROPRIATE | NOT-APPROPRIATE | NOT-APPROPRIATE |
| 15. Were the results internally consistent? | YES | YES | YES | YES | YES | YES | YES | YES |
| 16. Were the results for the analyses described in the methods, presented? | YES | YES | YES | YES | YES | YES | YES | YES |

| **The Appraisal tool for Cross-Sectional Studies (AXIS tool, 2016) Part 5** | | | | | | | | |
| --- | --- | --- | --- | --- | --- | --- | --- | --- |
| Study | **Lingenfelser et al., 1994** | **Maltese et al., 2016** | **Nguyen et al., 2012** | **Niu et al., 2013** | **Ohlinger et al., 2011** | **Orton and Gruzelier, 1989** | **Ovaskainen and Heikkilä, 2007** | **Özdemir et al., 2013** |
| Introduction |  |  |  |  |  |  |  |  |
| 1. Were the aims/objectives of the study clear? | YES | YES | YES | YES | YES | YES | YES | YES |
| Methods |  |  |  |  |  |  |  |  |
| 2. Was the study design appropriate for the stated aim(s)? | YES | YES | YES | YES | YES | YES | YES | YES |
| 3. Was the sample size justified? | NO | NO | YES | YES | NO | NO | NO | NO |
| 4. Was the target/reference population clearly defined? (Is it clear who the research was about?) | YES | YES | YES | YES | YES | YES | YES | YES |
| 5. Was the sample frame taken from an appropriate population base so that it closely represented the target/reference population under investigation? | YES | YES | YES | YES | NO | YES | YES | YES |
| 6. Was the selection process likely to select subjects/participants that were representative of the target/reference population under investigation? | YES | YES | YES | YES | YES | YES | YES | YES |
| 7. Were measures undertaken to address and categorise non-responders? | NO | NO | NO | NO | NO | NO | NO | NO |
| 8. Were the risk factor and outcome variables measured appropriate to the aims of the study? | NO | YES | YES | YES | YES | NO | NO | NO |
| 9. Were the risk factor and outcome variables measured correctly using instruments/ measurements that had been trialled, piloted or published previously? | YNO | YES | YES | YES | YES | NO | NO | NO |
| 10. Is it clear what was used to determined statistical significance and/or precision estimates? (eg, p values, CIs) | YES | YES | YES | YES | YES | YES | YES | YES |
| 11. Were the methods (including statistical methods) sufficiently described to enable them to be repeated? | YES | YES | YES | YES | YES | YES | YES | YES |
| Results |  |  |  |  |  |  |  |  |
| 12. Were the basic data adequately described? | YES | YES | YES | YES | YES | YES | YES | YES |
| 13. Does the response rate raise concerns about non-response bias? | NO | NO | NO | NO | NO | NO | NO | NO |
| 14. If appropriate, was information about non-responders described? | NOT-APPROPRIATE | NOT-APPROPRIATE | NOT-APPROPRIATE | NOT-APPROPRIATE | NOT-APPROPRIATE | NOT-APPROPRIATE | NOT-APPROPRIATE | NOT-APPROPRIATE |
| 15. Were the results internally consistent? | YES | YES | YES | YES | YES | YES | YES | YES |
| 16. Were the results for the analyses described in the methods, presented? | YES | YES | YES | YES | YES | YES | YES | YES |

| Study | **Peng et al., 2022** | **Persico et al., 2018** | **Petru et al., 2005** | **Prasad et al., 2021** | **Proctor et al., 1996** | **Rouch et al., 2005** | **Russell et al., 2016** |
| --- | --- | --- | --- | --- | --- | --- | --- |
| Introduction |  |  |  |  |  |  |  |
| 1. Were the aims/objectives of the study clear? | YES | YES | YES | YES | YES | YES | YES |
| Methods |  |  |  |  |  |  |  |
| 2. Was the study design appropriate for the stated aim(s)? | YES | YES | YES | YES | YES | YES | YES |
| 3. Was the sample size justified? | NO | NO | YES | YES | NO | NO | NO |
| 4. Was the target/reference population clearly defined? (Is it clear who the research was about?) | YES | YES | YES | YES | YES | YES | YES |
| 5. Was the sample frame taken from an appropriate population base so that it closely represented the target/reference population under investigation? | NO | NO | YES | YES | YES | YES | NO |
| 6. Was the selection process likely to select subjects/participants that were representative of the target/reference population under investigation? | YES | YES | YES | YES | YES | YES | YES |
| 7. Were measures undertaken to address and categorise non-responders? | NO | NO | NO | NO | NO | YES | NO |
| 8. Were the risk factor and outcome variables measured appropriate to the aims of the study? | YES | YES | NO | NO | YES | YES | NO |
| 9. Were the risk factor and outcome variables measured correctly using instruments/ measurements that had been trialled, piloted or published previously? | YES | YES | NO | NO | YES | YES | NO |
| 10. Is it clear what was used to determined statistical significance and/or precision estimates? (eg, p values, CIs) | YES | YES | YES | YES | YES | YES | YES |
| 11. Were the methods (including statistical methods) sufficiently described to enable them to be repeated? | YES | YES | YES | YES | YES | YES | YES |
| Results |  |  |  |  |  |  |  |
| 12. Were the basic data adequately described? | YES | YES | YES | YES | YES | YES | YES |
| 13. Does the response rate raise concerns about non-response bias? | NO | NO | NO | NO | NO | NO | NO |
| 14. If appropriate, was information about non-responders described? | NOT-APPROPRIATE | NOT-APPROPRIATE | NOT-APPROPRIATE | NOT-APPROPRIATE | NOT-APPROPRIATE | NO | NOT-APPROPRIATE |
| 15. Were the results internally consistent? | YES | YES | YES | YES | YES | YES | YES |
| 16. Were the results for the analyses described in the methods, presented? | YES | YES | YES | YES | YES | YES | YES |

| **The Appraisal tool for Cross-Sectional Studies (AXIS tool, 2016) Part 7** | | | | | | | | |
| --- | --- | --- | --- | --- | --- | --- | --- | --- |
| Study | **Saricaoğlu et al., 2005** | **Shwetha and Sudhakar, 2012** | **Shwetha and Sudhakar, 2014** | **Smith et al., 1995** | **Soares and de Almondes, 2017** | **Stout et al., 2021** | **Sun et al., 2021** | **Taylor et al., 2019** |
| Introduction |  |  |  |  |  |  |  |  |
| 1. Were the aims/objectives of the study clear? | YES | YES | YES | YES | YES | YES | YES | YES |
| Methods |  |  |  |  |  |  |  |  |
| 2. Was the study design appropriate for the stated aim(s)? | YES | YES | YES | YES | YES | YES | YES | YES |
| 3. Was the sample size justified? | NO | NO | NO | NO | NO | NO | NO | NO |
| 4. Was the target/reference population clearly defined? (Is it clear who the research was about?) | YES | YES | YES | YES | YES | YES | YES | YES |
| 5. Was the sample frame taken from an appropriate population base so that it closely represented the target/reference population under investigation? | NO | NO | NO | NO | NO | YES | YES | NO |
| 6. Was the selection process likely to select subjects/participants that were representative of the target/reference population under investigation? | YES | YES | YES | YES | YES | YES | YES | YES |
| 7. Were measures undertaken to address and categorise non-responders? | NO | NO | NO | NO | NO | NO | YES | NO |
| 8. Were the risk factor and outcome variables measured appropriate to the aims of the study? | NO | NO | NO | NO | NO | YES | YES | NO |
| 9. Were the risk factor and outcome variables measured correctly using instruments/ measurements that had been trialled, piloted or published previously? | NO | NO | NO | NO | NO | YES | YES | NO |
| 10. Is it clear what was used to determined statistical significance and/or precision estimates? (eg, p values, CIs) | YES | YES | YES | YES | YES | YES | YES | NO |
| 11. Were the methods (including statistical methods) sufficiently described to enable them to be repeated? | YES | YES | YES | YES | YES | YES | YES | NO |
| Results |  |  |  |  |  |  |  |  |
| 12. Were the basic data adequately described? | YES | YES | YES | YES | YES | YES | YES | YES |
| 13. Does the response rate raise concerns about non-response bias? | NO | NO | NO | NO | NO | NO | NO | NO |
| 14. If appropriate, was information about non-responders described? | NOT-APPROPRIATE | NOT-APPROPRIATE | NOT-APPROPRIATE | NOT-APPROPRIATE | NOT-APPROPRIATE | NOT-APPROPRIATE | NOT-APPROPRIATE | NOT-APPROPRIATE |
| 15. Were the results internally consistent? | YES | YES | YES | YES | YES | YES | YES | YES |
| 16. Were the results for the analyses described in the methods, presented? | YES | YES | YES | YES | YES | YES | YES | YES |

| **The Appraisal tool for Cross-Sectional Studies (AXIS tool, 2016) Part 8** | | | | | | | | | | |
| --- | --- | --- | --- | --- | --- | --- | --- | --- | --- | --- |
| Study | **Titova et al., 2016** | **Vajravelu et al., 2016** | **Van Der Linden et al., 2005** | **Veddeng et al., 2014** | **Virtanen et al., 2009** | **Vuori et al., 2014** | **Wagner-Hartl et al., 2018** | **Williams et al., 2017** | **Zhao et al., 2021** | **Zhuo et al., 2021** |
| Introduction |  |  |  |  |  |  |  |  |  |  |
| 1. Were the aims/objectives of the study clear? | YES | YES | YES | YES | YES | YES | YES | YES | YES | YES |
| Methods |  |  |  |  |  |  |  |  |  |  |
| 2. Was the study design appropriate for the stated aim(s)? | YES | YES | YES | YES | YES | YES | YES | YES | YES | YES |
| 3. Was the sample size justified? | NO | NO | NO | NO | NO | NO | NO | YES | NO | NO |
| 4. Was the target/reference population clearly defined? (Is it clear who the research was about?) | YES | YES | YES | YES | YES | YES | YES | YES | YES | YES |
| 5. Was the sample frame taken from an appropriate population base so that it closely represented the target/reference population under investigation? | YES | NO | NO | NO | YES | YES | NO | YES | YES | YES |
| 6. Was the selection process likely to select subjects/participants that were representative of the target/reference population under investigation? | YES | YES | NO | YES | YES | YES | YES | YES | YES | YES |
| 7. Were measures undertaken to address and categorise non-responders? | NO | NO | NO | NO | YES | YES | YES | NO | NO | YES |
| 8. Were the risk factor and outcome variables measured appropriate to the aims of the study? | YES | YES | NO | NO | YES | YES | NO | YES | NO | YES |
| 9. Were the risk factor and outcome variables measured correctly using instruments/ measurements that had been trialled, piloted or published previously? | YES | YES | NO | NO | YES | YES | NO | YES | NO | YES |
| 10. Is it clear what was used to determined statistical significance and/or precision estimates? (eg, p values, CIs) | YES | YES | YES | NO | YES | YES | YES | YES | YES | YES |
| 11. Were the methods (including statistical methods) sufficiently described to enable them to be repeated? | YES | YES | YES | NO | YES | YES | YES | YES | YES | YES |
| Results |  |  |  |  |  |  |  |  |  |  |
| 12. Were the basic data adequately described? | YES | YES | YES | YES | YES | YES | YES | YES | YES | YES |
| 13. Does the response rate raise concerns about non-response bias? | NO | NO | NO | NO | NO | NO | NO | NO | NO | NO |
| 14. If appropriate, was information about non-responders described? | NOT-APPROPRIATE | NOT-APPROPRIATE | NOT-APPROPRIATE | NOT-APPROPRIATE | YES | YES | NOT-APPROPRIATE | NO | NOT-APPROPRIATE | YES |
| 15. Were the results internally consistent? | YES | YES | YES | YES | YES | YES | YES | YES | YES | YES |
| 16. Were the results for the analyses described in the methods, presented? | YES | YES | YES | YES | YES | YES | YES | YES | YES | YES |
